# Yet Another Compact Time Series Data Representation Using CBOR Templates (YACTS)

**DOI:** 10.3390/s23115124

**Published:** 2023-05-27

**Authors:** Sebastian Molina Araque, Ivan Martinez, Georgios Z. Papadopoulos, Nicolas Montavont, Laurent Toutain

**Affiliations:** 1 IMT Atlantique Campus Rennes, SRCD, IRISA, 35510 Brest, France; juan.molina-araque@imt-atlantique.fr (S.M.A.); nicolas.montavont@imt-atlantique.fr (N.M.); laurent.toutain@imt-atlantique.fr (L.T.); 2 Nokia Bell Labs, 91300 Massy, France; ivan.martinez_bolivar@nokia-bell-labs.com

**Keywords:** Internet of Things (IoT), Time Series (TS), interoperability, CBOR, JSON, Protobuf, ASN.1

## Abstract

The Internet of Things (IoT) technology is growing rapidly, while the IoT devices are being deployed massively. However, interoperability with information systems remains a major challenge for this accelerated device deployment. Furthermore, most of the time, IoT information is presented as Time Series (TS), and while the majority of the studies in the literature focus on the prediction, compression, or processing of TS, no standardized representation format has emerged. Moreover, apart from interoperability, IoT networks contain multiple constrained devices which are designed with limitations, e.g., processing power, memory, or battery life. Therefore, in order to reduce the interoperability challenges and increase the lifetime of IoT devices, this article introduces a new format for TS based on CBOR. The format exploits the compactness of CBOR by leveraging delta values to represent measurements, employing tags to represent variables, and utilizing templates to convert the TS data representation into the appropriate format for the cloud-based application. Moreover, we introduce a new refined and structured metadata to represent additional information for the measurements, then we provide a Concise Data Definition Language (CDDL) code to validate the CBOR structures against our proposal, and finally, we present a detailed performance evaluation to validate the adaptability and the extensibility of our approach. Our performance evaluation results show that the actual data sent by IoT devices can be reduced by between 88% and 94% compared to JavaScript Object Notation (JSON), between 82% and 91% compared to Concise Binary Object Representation (CBOR) and ASN.1, and between 60% and 88% compared to Protocol buffers. At the same time, it can reduce Time-on-Air by between 84% and 94% when a Low Power Wide Area Networks (LPWAN) technology such as LoRaWAN is employed, leading to a 12-fold increase in battery life compared to CBOR format or between a 9-fold and 16-fold increase when compared to Protocol buffers and ASN.1, respectively. In addition, the proposed metadata represent an additional 0.5% of the overall data transmitted in cases where networks such as LPWAN or Wi-Fi are employed. Finally, the proposed template and data format provide a compact representation of TS that can significantly reduce the amount of data transmitted containing the same information, extend the battery life of IoT devices, and improve their lifetime. Moreover, the results show that the proposed approach is effective for different data types and it can be integrated seamlessly into existing IoT systems.

## 1. Introduction

The Internet of Things (IoT) has emerged as one of the most transformative technologies of recent years, having extended the reach of the internet beyond traditional computer networks [1]. It encompasses all kinds of devices and objects that can transmit or receive digital data, and has garnered the attention of academia and industry due to its rapid growth [2,3]. A clear indicator of the IoT expansion is the surge in Machine-to-Machine (M2M) connections, which has grown from approximately one billion in 2017 to an estimated 3.9 billion by 2022. Furthermore, other studies predict that the number of IoT devices will escalate to 22 billion by 2025 and 50 billion by the end of the decade [1,4,5,6].

Despite this significant growth, the increasing demand for IoT connectivity and the diverse requirements of various applications have made it increasingly challenging to develop cost-effective, adaptable, and intelligently efficient IoT systems within the context of a massive-IoT paradigm [6]. In addition, the scale of IoT deployment and the substantial volume of data collected by IoT devices make their integration into an information system challenging. Currently, the selection and incorporation of devices into IoT systems occur during the conception phase, which means that modifying an application may necessitate replacing the device. Conversely, the introduction of new devices in the network may require the modification of the application to support them. This leads to a strong dependency between cloud applications and IoT devices.

**Figure 1:**
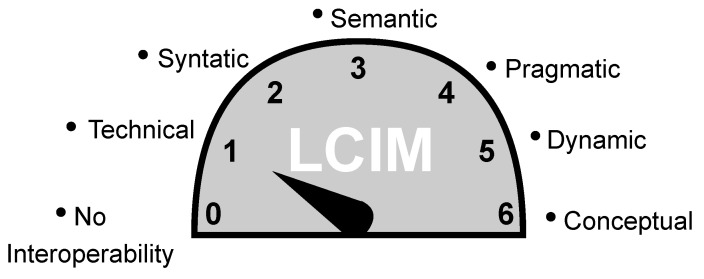
Levels of interoperability by Tolk and Muguira in [8].

Furthermore, in the scenario of an increasing number of distributed and heterogeneous IoT devices, there is a demand for technologies that support interoperability, providing means of representation, discovery, and integration [7]. To manage the interoperability-related issues, IoT devices need to be compatible with each other, which requires the use of common communication protocols and standards. Tolk and Muguira have defined different levels of interoperability in [8] and are illustrated in Figure 1. Thus, while wireless standards have resolved a number of connectivity-related issues, interoperability-related challenges still exist. For instance, even if devices use the same communication protocols or standards, they may use different syntaxes for representing data, which can cause errors or misinterpretations. Next, if IoT devices use the same communication protocols and data formats, they can interpret the data differently based on their individual context or understanding depending on the application or network. Finally, it is also possible that different organizations may use different standards, policies, or processes which can affect how they integrate IoT devices into their operations [9,10,11,12].

In addition to the interoperability challenges, a typical IoT system architecture consists of multiple constrained devices that are connected to aggregating gateways. Then, the gateways are linked to cloud platforms to deliver the information sensed by the constrained devices [13]. However, constrained devices are commonly designed with cost and power efficiency as primary considerations, resulting in highly constrained resources in terms of power, memory, and processing capabilities [14]. In particular, sensors function through battery energy, and this energy is used up when the sensor transmits data. The better the quality and quantity of the information transmitted, the more accurately the sensor can monitor the environment. However, this also means that the battery is drained more rapidly. Because of these limitations in energy supply, it is crucial to create efficient plans for controlling sensor transmissions that find a suitable compromise between monitoring precision and energy consumption [15].

In this article, we introduce the Variable-based TS (VTS) template, a versatile template with a specific representation format for Time Series (TS) based on CBOR [16]. This compact format and template constitute the starting point for decoupling the application data format from the sensor data format. Our proposal employs the characteristics of the CBOR to depict measurements by utilizing the difference between subsequent values in the TS (delta), while using a hierarchical tree-like structure encoded in CBOR with a Tag Number (TAGN) to determine the location of the values within the TS. Moreover, metadata are utilized to depict supplementary information such as the sensor precision, time stamp, and sensor identifiers.

In [17], we introduced a new and versatile format for standardizing the representation of time series data, based on CBOR. This format is highly efficient and aims to separate the format of sensor data from the application. With this approach, we aim to provide a more flexible and adaptable way of handling TS data. Moreover, we have also presented a novel architecture that allows for the transformation of this representation into any data format that is required by any cloud application. By decoupling the representation format from the application data format, our architecture enables the conversion of the TS data into different formats with ease, providing greater flexibility and interoperability between different systems. This article extends [17] with the following contributions:

- A new refined and structured metadata to provide a more comprehensive and organized representation of additional information such as sensor precision, time stamp, and sensor identifiers;
- A comparison between the proposed metadata and the format used in [17];
- A CDDL code to validate CBOR structures against the proposed VTS template, facilitating the identification of malformed CBOR structures;
- A performance evaluation with different data types (i.e., Global Positioning System (GPS) measurements) to validate the adaptability of our approach;
- A performance evaluation of our proposal against the most popular data representations: JSON, CBOR, ASN.1, and Protocol buffers. It considers:
  – IoT devices’ battery life;
  – LoRaWAN transmissions’ Time-on-Air;
  – Packet Fragmentation;
  – Overhead of protocol headers: LoRaWAN and SCHC (UDP/IP/CoAP).

The remainder of this article is organized as follows: in Section 2 we provide an overview describing the most prevalent formats used for representing information on the internet, as well as the related work present nowadays. Then, Section 3 outlines the problem statement to further introduce the IoT architecture that facilitates the seamless integration of IoT devices into diverse information systems in Section 4. Section 5 presents our Variable-based TS (VTS) template along with its detailed metadata and the data format proposal, which reduces the amount of data sent by IoT devices while preserving the same information. Furthermore, the VTS template and the data format proposed will enable the translation of the transmitted data format into any required data format compatible with the cloud application in an IoT network.

Eventually, this article continues with the performance evaluation, which demonstrates that the actual data sent by IoT devices can be reduced by between 88% and 94% when compared against JSON, and between 64% and 91% when compared against binary formats as CBOR, ASN.1 or Protocol buffers. Additionally, it reduces between 80% and 96% the Time-on-Air leading to an incrementation of a 12-fold increase in battery life compared to Protocol buffers, ASN.1, CBOR and JSON formats. Moreover, in cases where networks such as LPWAN or Wi-Fi are employed, the metadata represents only an additional 0.5% of the overall data transmitted. This implies that it is possible to transmit the context for all the measurements using a small number of bytes. Later, in Section 7 we present a brief discussion about the benefits, drawbacks, and challenges of our proposal. Finally, in Section 8 we present our conclusions based on our findings and analysis.

## 2. Technical Background and Related Work

This section aims to provide a description of the most commonly employed representation formats on the internet and the related work present in the literature. To start, we will first review JSON [18], a widely adopted data representation format that is known for its ease of use and human-readable structure. Then, we will delve into CBOR [16], a binary data format that has proven to be a fundamental aspect of our research. After, we will present an overview of SenML [19], a format that is specifically designed to facilitate the representation of sensor measurements and device parameters in a systematic and straightforward manner. Then, we will provide a summary of RDF [20], a widely used standardized model for exchanging data over the web, based on syntax notations. Later, we will examine Concise Data Definition Language (CDDL) [21]; this language has different roles as it serves as an extension to CBOR and define different data structures. Finally, we present a literature review of different binary formats commonly used for data representation on the internet [22,23,24,25,26,27].

### 2.1. JSON

JSON, as defined in [18], is a data interchange format that establishes a comprehensive set of rules for the organization and serialization of structured data. This format has gained widespread popularity and acceptance among developers, particularly on the internet, due to its versatility and ease of use. The major advantages of using JSON include:

- JSON is easily comprehended and written by humans, allowing for efficient and intuitive data manipulation.
- JSON is a compact data format in comparison to other alternatives such as XML, resulting in reduced data transmission time and storage requirements.
- JSON employs conventions commonly utilized in programming languages, while maintaining language independence, and it enables seamless integration with a wide range of other programming languages and platforms.

The JSON notation is built on a collection of “name/value” pairs, where each “name” is a string and the “value” can be either an object ({}), an array ([]), a string, a number, or values as ’true’, ’false’, or ’null’. This structure allows for the representation of complex data structures, such as nested objects and arrays, in a clear and organized manner. Additionally, JSON also allows for the representation of ordered lists of values, enabling the efficient representation of data sequences such as Time Series data [18]. Listing 1 shows an example of a JSON structure.

**Listing 1.** A JSON example that consists of four sensor measurements. Three for ‘/current’ measurements and one for ‘/voltage’ measurements. The sensor is identified by the following URL ‘urn:dev:mac:0024befffe804ff1/’.

[

    { ‘sensor’:     ‘urn:dev:mac:0024befffe804ff1/current’,

      ‘time’:      ‘Tuesday 8 June 2010 18:01:16’,

      ‘unit’:      ‘A’,

      ‘value’:      1.7

      },

    { ‘sensor’:     ‘urn:dev:mac:0024befffe804ff1/current’,

      ‘time’:      ‘Tuesday 8 June 2010 18:01:15’,

      ‘unit’:      ‘A’,

      ‘value’:      1.6

      },

    { ‘sensor’:     ‘urn:dev:mac:0024befffe804ff1/current’,

      ‘time’:      ‘Tuesday 8 June 2010 18:01:14’,

      ‘unit’:      ‘A’,

      ‘value’:      1.5

      },

    { ‘sensor’:     ‘urn:dev:mac:0024befffe804ff1/voltage’,

      ‘time’:      ‘Tuesday 8 June 2010 18:01:16’,

      ‘unit’:      ‘V’,

      ‘value’:      120.1

      }

]



Listing 1 represents four sensor measurements, three for current and one for voltage, where each one is represented in an object ({}) with four key-value pairs:

- The first key-value pair are strings (“sensor”: “urn:dev:mac:0024befffe804ff1/current”), and indicates the identifier for the sensor measurement.
- The second key-value pair are strings (“time”: “Tuesday 8 June 2010 18:01:15”), and indicates the time when the measurement was taken by the sensor.
- The third key-value pair are also strings (“unit”: “A” or “unit”: “V”), which indicates the unit for the measurements, Amperes (A) or Voltage (V).
- The fourth key-value pair is composed of the string ‘value’, and a float number indicating the value for each measurement (i.e., 1.5, 1.6, 1.7, and 120.1).

Furthermore, JSON also has a number of additional features that make it a versatile and a powerful data interchange format. JSON is easy to parse and generate by machine, allowing for efficient data manipulation and automation. It is also lightweight and can be transmitted over a network without the need for additional software or libraries. Finally, JSON is self-describing and human-readable, making it easy to debug and troubleshoot.

In conclusion, JSON is a widely adopted data interchange format that offers a comprehensive set of rules for the organization and serialization of structured data. Its versatility, ease of use, and language independence make it a popular choice for developers working on a wide range of projects. JSON is a powerful tool for data representation and storage that allows for the efficient and intuitive manipulation of data.

### 2.2. Concise Binary Object Representation (CBOR)

CBOR is a data serialization format that is designed to be more efficient and compact than JSON. This format is particularly well-suited for IoT applications, where the need for efficient data transfer and storage is crucial. The CBOR format was designed by the Internet Engineering Task Force (IETF) and is specified in [16] including the following goals:

1. Represent common internet standard data formats without ambiguity: This ensures that the data can be easily understood and interpreted by any system that uses CBOR.
2. Have a compact code for encoding and decoding: CBOR has been designed to have a compact codebase, which makes it easy to implement and use in various systems. This allows for faster processing times and reduces the resources required for encoding and decoding data.
3. Allow data to be decoded without a schema description: CBOR is designed to be self-describing, which means that data can be decoded without the need for a schema description. This allows for greater flexibility and ease of use, as it eliminates the need to define and maintain a separate schema.
4. Be reasonably compact in terms of serialization: CBOR has been optimized for compactness, which means that the serialized data are smaller in size compared to other binary data formats. This makes it more efficient for use in high-volume applications and for transmission over constrained networks.
5. Can be applied to both constrained nodes and high-volume applications: CBOR is designed to be versatile, which means that it can be used in a wide range of applications, from high-volume applications that require fast processing times to constrained nodes that have limited resources.
6. Support all JSON data types: CBOR is designed to be compatible with JSON, which means that it supports all of the data types used in JSON. This allows for interoperability between systems that use CBOR and those that use JSON.
7. Be extensible in terms of format: CBOR has been designed to be extensible, which means that new data types and features can be added to the format as needed. This allows for future updates and improvements to the format and ensures that it stays relevant and useful for a long time.

CBOR is a self-describing format, such as JSON, which makes it easy to read and understand. However, it uses a binary representation instead of a text-based representation, making it more efficient for data transmission and storage. Additionally, CBOR supports a wider range of data types than JSON, including integers of various sizes, floating-point numbers, binary data, maps, and arrays. As a result, CBOR is able to represent data in a more compact form than JSON. Table 1 presents the different data types supported by CBOR, along with examples of their representation.

When the size of messages serialized using CBOR and JSON were compared in earlier studies, the findings showed that CBOR had a substantially more compact representation in terms of bytes, with an approximate 26% reduction in size when compared to JSON [28]. For example, in JSON, a large integer would be represented as a string, whereas in CBOR it can be represented in a more compact binary format. To illustrate this, consider Listing 1 from Section 2.1, it requires 447 when represented in JSON, while with CBOR, it would require 407 bytes, as it can be observed in Listing 2.

It is important to note that the reduction presented in Listing 2 does not utilize the optimal encoding for floating numbers in the CBOR format. Specifically, the encoding of the number 120.1 requires 4 bytes in JSON, but up to 9 bytes in CBOR. Additionally, the representation of strings in CBOR is not efficient compared to JSON, as it requires the same number of bytes in both formats. CBOR also supports a feature called “tags”, which can be used to attach additional information to data types. For example, a timestamp could be represented as a CBOR tag, indicating that the data are timestamps. This feature allows for added flexibility and extensibility in the representation of data.

Overall, CBOR is a highly efficient, compact, and extensible data serialization format, which can be used for various applications, especially for IoT, where the need for efficient data transfer and storage is crucial. Its ability to represent data in a more compact form, support a wider range of data types, and add flexibility through the use of tags and indefinite-length data make it a valuable alternative to JSON in IoT applications.

**Listing 2.** The CBOR representation of Listing 2 in hexadecimal. It starts with an array of four objects (84); then, for each object, there is a map with four key-value pairs (A4), i.e., (i) “sensor”: “urn:dev.../current”, (ii) “time”: “Tue... 18:01:16”, (iii) “unit”: “A”, and iv) “value”: 1.7. For example, the CBOR encoding 66 (i.e., line 3) is indicating the type of the variable (i.e., first key from the first key-value pair) which is a string in this example that consists of 6 characters, i.e., “sensor”. While the second key from the first key-value pair is encoded with 78 24 (i.e., line 5), and it indicates that the variable is a string of 36 characters, i.e., “urn:dev.../ current”. Another example would be the last key-value pair, where we have the CBOR encoding 65 (i.e., line 15) which indicates that the variable is a string of 5 characters, i.e., “value”, while the second key from the last key-value pair is encoded with FB 3FFB333333333333 (i.e., line 17), and it indicates the floating number of 1.7.

1.  84                         # array(4)

2.     A4                      # map(4)

3.        66                   # text(6)

4.          73656E736F72           # "sensor"

5.        78 24                 # text(36)

6.          75726E3A6... F63757272656E74  # "urn:dev.../current"

7.        64                   # text(4)

8.          74696D65              # "time"

9.        78 1C                 # text(28)

10.         54756573646179... 31383A3036  # "Tue... 18:01:16"

11.       64                   # text(4)

12.         756E6974              # "unit"

13.       61                   # text(1)

14.         41                 # "A"

15.       65                   # text(5)

16.         76616C7565             # "value"

17.       FB 3FFB333333333333         # 1.7

18.    A4                      # map(4)

19.      ...

20.      ...

21.    A4                      # map(4)

22.       66                   # text(6)

23.         73656E736F72           # "sensor"

24.       78 24                 # text(36)

25.         75726E3A6D6... 66F6C74616765  # "urn:dev.../voltage"

26.       64                   # text(4)

27.         74696D65              # "time"

28.       78 1C                 # text(28)

29.         5475657364617... A30313A3136  # "Tue... 18:01:16"

30.       64                   # text(4)

31.         756E6974              # "unit"

32.       61                   # text(1)

33.         56                 # "V"

34.       65                   # text(5)

35.         76616C7565            # "value"

36.       FB 405E066666666666         # 120.1



### 2.3. Sensor Measurement List (SenML)

Sensor Measurement List (SenML), is a data format for representing sensor measurements and metadata in a structured way. It is proposed in [19] as a way to standardize the representation of sensor data in the IoT ecosystem. The main goals of SenML were to provide a lightweight, flexible, and extensible format for representing sensor data, as well as to facilitate the integration of sensor data with existing web technologies.

SenML uses a simple, hierarchical structure to represent sensor data, where every sensor reading is represented as an element within the hierarchy. Each element can include a variety of metadata, such as the type of sensor, the units of measurement, and the time the measurement was taken. This allows for easy interpretation of the data by both humans and machines. For instance, Listing 3 represents a temperature measurement in Celsius degrees with JSON syntax.

**Listing 3.** A single SenML Record example. More specifically, ‘n’ stands for the name (or the identification) of the sensor, and it comes with the value ‘urn:dev:ow:10e2073a01080063’. Then, the unit is represented with ‘u’ and the value ‘Cel’ for Celsius degrees. Finally, the value is represented with ‘v’ that in this example comes with the value 23.1.

[

    {  ‘n’: ‘urn:dev:ow:10e2073a01080063’,

        ‘u’: ‘Cel’,‘v’:23.1  }

]



One of the key characteristics of SenML is its capability to encapsulate multiple measurements in a single message, thereby enabling efficient transmission of sensor data and the correlation of measurements from various sensors. This feature allows for the compact representation of sensor data, reducing the amount of data that needs to be transmitted, and making it easier to correlate data from different sensors.

SenML also facilitates the usage of ‘base values’ when representing multiple measurements in a single message. This allows for optimization and reusability of values per measurement, which can further enhance the efficiency of sensor data transmission. This feature allows to reduce the size of the message by not repeating the same values for each measurement, which can be especially useful in scenarios where a large number of measurements need to be transmitted frequently. An example employing the concept of ‘base values’, specifically ‘base name (bn)’, ‘base unit (bu)’, and ‘base time (bt)’, is depicted in Listing 4. In this context, the example shows Listing 1 from Section 2.1 employing the SenML data format. Then, Listing 4 serves as an illustration of how SenML can be utilized to represent data in a structured manner. In this case, with the use of ‘base values’ is possible to eliminate the need to repeatedly send specific information, such as the sensor identification (i.e., ’urn:dev:mac:0024befffe804ff1/’), the time (i.e., ‘bt’: 1276020076 represented in EPOCH format), and the unit for the current (i.e., ’A’). Thus, the use of ’base values’ enables the transmission of only the necessary information, making the data transfer process more compact.

**Listing 4.** The SenML representation of the Listing 1. The first object only contains base values which will apply to all the other objects. Then the identifier of the sensor is depicted with the values of ‘bn’+‘n’ (i.e., ‘urn:dev:mac:0024befffe804ff1/’+‘current’ or ‘urn:dev:mac:0024befffe804ff1/’+‘voltage’). Then, the time is represented with the values of ‘bt’+‘t’, for example, 1276020076 in EPOCH format plus −2 which is equal to 1276020074. Finally, the unit of the object that contains ‘voltage’ overrides the ‘bu’: ‘A’ with ‘u’:‘V’, where V stands for Voltage.

[

    { ‘bn’: ‘urn:dev:mac:0024befffe804ff1/’,

      ‘bt’: 1276020076,           ‘bu’: ‘A’},

    { ‘n’: ‘voltage’,    ‘u’:‘V’, ‘v’:  120.1 },

    { ‘n’: ‘current’,    ‘t’:-2,   ‘v’:  1.5 },

    { ‘n’: ‘current’,    ‘t’:-1,   ‘v’:  1.6 },

    { ‘n’: ‘current’,    ‘t’:0,   ‘v’:  1.7 }

]



Finally, SenML is supported by several data formats including JSON, CBOR, Extensible Markup Language (XML), and Efficient XML Interchange (EXI). These formats are able to share the common SenML data model, allowing for the representation of sensor data in a format that is easy to parse and generate.

### 2.4. Resource Description Framework (RDF)

Resource Description Framework (RDF) is a standardized data model for representing and linking data on the web developed by the World Wide Web Consortium (W3C). It is a way of structuring data in a graph format, where nodes represent resources (e.g., sensors, actuators, or things) and edges represent relationships between them [20].

RDF is particularly useful for IoT applications, as it enables the integration of heterogeneous data from a wide range of sources. This is important in IoT, where data are often generated by different devices, using different protocols and formats. By using RDF, data can be integrated and queried in a uniform way, regardless of their source [29,30].

Then, in RDF the information is represented in triplets. A triple consists of three parts: subject, predicate, and object. These three parts are used to represent statements about resources in a graph format [20]:

- Subject: The subject is the resource that the statement is about. It is represented by an Uniform Resource Identifier (URI) or a blank node. The subject is usually the first element of an RDF triple and describes the resource being talked about [20].
- Predicate: The predicate is the property or relationship being asserted about the subject. It is represented by the URI that identifies the property. The predicate is usually the second element of an RDF triple [20].
- Object: The object is the value of the property or relationship. It can be a literal (e.g., a string or a number) or another resource (identified by a URI or a blank node). The object is usually the third element of an RDF triple [20].

At the same time, this data model can be represented as turtle syntax or using different languages as XML or JSON for Linking Data (JSON-LD). For instance, Listings 5–7 depict an example of RDF/JSON-LD, turtle syntax and RDF/XML respectively [30].

**Listing 5.** The RDF turtle representation of the Listing 1. In this example, urn:dev:mac:0024befffe804ff1/current and urn:dev:mac:0024befffe804ff1/voltage are represented as instances of the http://.../Sensor class. Each measurement for a sensor is represented as a blank node (_:m1, _:m2, etc.) connected to the corresponding sensor with the http://.../hasMeasurement property. The time, unit, and value of each measurement are represented as properties of the blank node using the URIs http://.../time, http://.../hasUnit, and http://.../hasValue, respectively. The value is represented as a typed literal with the xsd:float data type.

@prefix rdf: <http://www.w3.org/1999/02/22-rdf-syntax-ns#>.

@prefix xsd: <http://www.w3.org/2001/XMLSchema#>.

 

<urn:dev:mac:0024befffe804ff1/current>

rdf:type <http://example.org/Sensor>.

 

<urn:dev:mac:0024befffe804ff1/voltage>

rdf:type <http://example.org/Sensor>.

 

<urn:dev:mac:0024befffe804ff1/current>

<http://example.org/hasMeasurement> _:m1.

_:m1 <http://example.org/time> "Tuesday 8 June 2010 18:01:16".

_:m1 <http://example.org/hasUnit> "A".

_:m1 <http://example.org/hasValue> "1.7"^^xsd:float.

         .

         .

         .

<urn:dev:mac:0024befffe804ff1/voltage>

<http://example.org/hasMeasurement> _:m4.

_:m4 <http://example.org/time> "Tuesday 8 June 2010 18:01:16".

_:m4 <http://example.org/hasUnit> "V".

_:m4 <http://example.org/hasValue> "120.1"^^xsd:float.



**Listing 6.** The RDF / JSON-LD representation of the Listing 1. Note that in RDF / JSON-LD, the @context is used to define the mapping between terms used in the JSON data and their corresponding URIs. The @graph contains an array of objects that represent the nodes and edges in the graph. The @id is used to identify the node, and the other properties correspond to the predicates and objects in the RDF triples.

{

  "@context": {

    "sosa": "http://www.w3.org/ns/sosa/",

    "time": "http://www.w3.org/2006/time#",

    "value": "sosa:hasSimpleResult",

    "unit": "sosa:hasUnit",

    "observation": "sosa:Observation",

    "sensor": "sosa:madeBySensor",

    "phenomenonTime": "sosa:phenomenonTime",

    "resultTime": "sosa:resultTime"

  },

  "@graph": [

    {

      "@id": "urn:dev:mac:0024befffe804ff1/current",

      "@type": "sosa:Observation",

      "sosa:madeBySensor": "urn:dev:mac:0024befffe804ff1",

      "sosa:phenomenonTime": "Tuesday 8 June 2010 18:01:16",

      "sosa:resultTime": "Tuesday 8 June 2010 18:01:16",

      "sosa:hasSimpleResult": 1.7,

      "sosa:hasUnit": "A"

    },

           .

           .

           .

    {

      "@id": "urn:dev:mac:0024befffe804ff1/voltage",

      "@type": "sosa:Observation",

      "sosa:madeBySensor": "urn:dev:mac:0024befffe804ff1",

      "sosa:phenomenonTime": "Tuesday 8 June 2010 18:01:16",

      "sosa:resultTime": "Tuesday 8 June 2010 18:01:16",

      "sosa:hasSimpleResult": 120.1,

      "sosa:hasUnit": "V"

    }

  ]

}



**Listing 7.** The RDF / XML representation of the Listing 1. Note that in RDF / XML, the rdf:about attribute is used to indicate the subject of a triple, and the properties (predicates) and values (objects) are nested inside the rdf:Description element.

<?xml version="1.0"?>

<rdf:RDF xmlns:rdf="http://www.w3.org/../../22-rdf-syntax-ns#"

     xmlns:ex="http://...#">


 


  <rdf:Description rdf:about="urn:::0024...804ff1/current">

    <ex:time>Tuesday 8 June 2010 18:01:16</ex:time>

    <ex:unit>A</ex:unit>

    <ex:value>1.7</ex:value>

  </rdf:Description>

        .

        .

        .

  <rdf:Description rdf:about="urn:::0024...804ff1/voltage">

    <ex:time>Tuesday 8 June 2010 18:01:16</ex:time>

    <ex:unit>V</ex:unit>

    <ex:value>120.1</ex:value>

  </rdf:Description>


 


</rdf:RDF>



Despite the fact that turtle syntax, RDF/XML, and RDF/JSON-LD representations can be advantageous for different IoT applications, these formats are too verbose for constrained devices with limited storage and processing power [30].

Moreover, in recent years, the research on this topic has focused on finding a format to compress large sets of RDF triples, with the aim of reducing network traffic. Two examples of such formats are HDT (Header, Dictionary, and Triples) [31] and RDSZ (RDF Differential Stream compressor based on Zlib) [32]. While these compression techniques have shown promising results in terms of decreasing network traffic, they are not suitable for very constrained devices, due to the processing power [30,33].

Then, the research in [30] proposed an approach that addresses the requirements for efficient serialization of RDF data in constrained devices. The proposed approach relies on the standardized W3Cs Efficient XML Interchange (EXI) format, which enables efficient serialization of RDF data and makes it applicable even for devices with limited capabilities. Thus, in [30], it is possible to convert from Listing 7 represented in RDF/XML to Listing 8. Thus, in Listing 8 the data are not repeated each time a measurement is described, instead, the elements that are repeated (e.g., strings as “unit”) are replaced by IDs.

**Listing 8.** The RDF / EXI representation of the Listing 7. The EXI coding mechanism used in (ref) is to assign a unique ID to the name of an unknown element or attribute when it is encountered for the first time in the stream (e.g., the unit is identified with ID = 1). This string-based name is then memorized and associated with the assigned ID. Subsequently, whenever the same string appears again in the stream, its assigned ID is used instead (e.g., 1 “V” in the listing).

00 "urn:::0024...804ff1/current"

  time "Tuesday 8 June 2010 18:01:16"

  unit "A"

  value "1.7"

  ...

04 "urn:::0024...804ff1/voltage"

  0 "Tuesday 8 June 2010 18:01:16"

  1 "V"

  2 "120.1"


 




### 2.5. Concise Data Definition Language (CDDL)

Concise Data Definition Language (CDDL) is a data definition language that is used to define data structures, developed by the Internet Engineering Task Force (IETF) [21]. The main goal of CDDL is to provide a simple and expressive way to define data structures that are both human-readable and machine-processable express for protocol messages and data formats that use CBOR or JSON structures. One of the key features of CDDL is its expressiveness, which enables the definition of complex data structures with minimal code. Additionally, it supports a wide range of data types, including integers, strings, and arrays, which makes it suitable for various applications. Furthermore, CDDL is designed to be extensible, allowing the addition of custom data types as needed. Moreover, CDDL allows to indicate the occurrence of a field, for example, whether it is mandatory or optional.

CDDL is used to define the data structures in CBOR or JSON. This allows for a compact and efficient representation of sensor data, making it easy to process and interpret. For example, the CDDL code in Listing 9 defines a sensor measurement with a name, unit, and value, and indicates that the name field is mandatory and the value field is optional.

**Listing 9.** A CDDL code example with different data types and occurrences. The object (i.e., Measurement) is represented by 3 key-value pairs, i.e., name, unit, and value for the keys, while the values are represented by the type of variable, tstr (i.e., text string) or float. The ‘+’ represents that the value name can be present at least one or more times, while ‘?’ indicates that this value is optional.

Measurement = {

    + name: tstr,

    unit: tstr,

    ? value: float,

}



As an illustration, consider that we generate the corresponding CDDL code to establish the SenML structure of Listing 4 in JSON format. The outcome is depicted in Listing 10, wherein we specify the fields of key-value pairs, the corresponding units, and the occurrence of each value.

**Listing 10.** The CDDL code that represents the structure of the Listing 4. SenMLexample is an array ([]) composed of two objects (i.e., baseValues, values). Moreover, the value 0*1 specifies an occurrence of 0 or 1 for each key-value pair.

SenMLexample = [ baseValues, values ]


 


baseValues = {  0*1 ‘bt’: number,       ; Base Time

          0*1 ‘bn’: tstr,        ; Base Name

          0*1 ‘bu’: tstr        ; Base Unit  }


 


values = {  0*1 ‘t’: int,         ; Time

        0*1 ‘n’: tstr,         ; Name

        0*1 ‘u’: tstr,         ; Unit

        0*1 ‘v’: float         ; Value  }



Thus, by utilizing the CDDL code along with the tools outlined in [21] enables the efficient generation and validation of data structures in both JSON and CBOR formats.

### 2.6. Literature Review on Binary Formats

Serialization is a process widely used in IoT to transmit information between devices. It involves converting data structures or object states into a format that can be transmitted over a network and then reconstructed on the other end. Thus, serialization is important for IoT devices as it allows for efficient and reliable transmission of data, which in turn can enhance the limited capabilities and prolong the lifespan of these devices [22]. Moreover, serialization formats are widely utilized for the efficient transmission of information between IoT devices [23]. Binary formats, a subset of serialization formats, represent data through binary encoding and are known for their ability to facilitate efficient transmission and storage, such as CBOR. Other binary formats identified in the literature include:

1. MessagePack: a schema-less open-source binary object serialization mechanism. It relies on Type System and Formats concepts, which define the data types supported, including key-value pairs, and how they are encoded. It is lightweight and has a small memory footprint, making it suitable for IoT devices [24]. Additionally, MessagePack is a widely implemented counted binary serialization format, similar in many properties to CBOR, although less strict in its definition and relies on implementation-specific details to determine how data should be encoded [16].
2. Binary JSON (BSON): an open-source, schema-less binary data serialization mechanism that was developed for the storage of key-value pairs (JSON-like maps) in the MongoDB database. It is designed to be lightweight and efficient and is represented as key-value pairs, where keys are strings and values can be any supported data type. Binary JSON (BSON) is suitable for IoT applications, particularly sensor nodes, due to its efficient encoding/decoding and fast traversal [24]. Its major distinguishing feature is the capability for an in-place update which allows it to maintain a compact representation. It can also be used in other applications through open-source libraries [16].
3. Abstract Syntax One (ASN.1): a standard for specifying the structure of data used in telecommunications and computer networking. It is used to define the syntax of messages exchanged between systems and provide a standard way of describing data exchanged between systems [16]. ASN.1 is independent of any specific programming language or implementation, and is widely used in communications such as Secure Sockets Layer (SSL)/Transport Layer Security (TLS) and the implementation of communication protocols such as Simple Network Management Protocol (SNMP), Lightweight Directory Access Protocol (LDAP), and others. It is often used in conjunction with the Basic Encoding Rules (BER) and other encoding rules such as the Distinguished Encoding Rules (DER) for specific applications [25].

In addition to the previously mentioned binary formats, there have been several other formats developed with various objectives. These objectives may not have been explicitly stated, but can be inferred from the context in which the format was first utilized. Examples of alternative binary formats with similar objectives include Protocol Buffers, designed by Google, PSON, Smile, and Message Services Data Transmission (MSDTP) [26,27].

## 3. Problem Statement

IoT device interoperability is a complicated system that needs to be considered from all angles. Therefore, a comprehensive solution is needed to address all interoperability issues, which have been identified as one of the most important problems of IoT [9]. To solve this interoperability problem, all IoT devices must be compatible with the devices they communicate with. This is only possible if they use common communication protocols and standards. The connectivity-related problems have been solved by wireless standards and most devices can communicate at the physical level, but there are some problems at the interoperability levels, i.e., technical, syntactic, semantic, and organizational.

In IoT environments, technical interoperability is still a major challenge, preventing up to 60% of potential economical benefits [34]. The main technological method to integrate numerous heterogeneous devices using different communication technologies is multimode radios. However, security concerns can arise when doing so. A device that uses multiple wireless protocols may provide more attack vectors through which malicious actors could secretly introduce unwanted malware and monitor network data [9].

For syntactic interoperability, messaging protocols such as Constrained Application Protocol (CoAP), Extensible Messaging and Presence Protocol (XMPP), Advanced Message Queuing Protocol (AMQP), and some platforms have emerged as solutions, each providing essential interoperability capabilities between domains. Although devices are capable of flawless communication, they still cannot understand each other. Therefore, additional techniques are required to express information semantics in a way that can be understood by all IoT devices. Thus, a consistent interpretation of semantic data in a globally shared ontology can be very helpful at the organizational level of interoperability. However, this is not always the case. Even though many local systems use well-accepted ontologies, they end up extending them and creating their own meanings and interfaces [9].

Even when interoperability is present, there is still the problem that Over the Top (OTT) companies do not want their products to be interoperable in order to gain a competitive advantage over customers. For this reason, they do not promote open Application Programming Interface (API) for their applications [10]. Furthermore, APIs related to IoT devices are mostly incompatible with each other and require a common API management system layer that can abstract the complexity of IoT devices [11].

The different devices with different data formats and different APIs are another major problem in IoT interoperability. Due to the lack of data semantics and common standards for interpreting the meaning of the data, as well as the incompatibility of devices at the data layer, the dynamic nature of the data is a significant barrier to communication between IoT devices. In addition, the data must be discoverable, which can be difficult with enormous volumes of remote networks and cloud-based data [11].

Finally, the majority of IoT data measurements are presented in TS format, nevertheless, this representation depends on the application or the environment in which the system is employed [12]. Even a single measurement is a degenerated case of a TS representation. Indeed, we have observed that the works from the literature are concentrated on exploiting the data rather than on efficiently representing it. For instance, in [35], the authors proposed a framework to improve the analysis of the information produced by sensor devices. This framework adds contextual and historical information to the initial data.

Next, in [36], the authors presented a new compression method of TS for IoT. Thus, the measurement reporting process was tailored to specific use cases in a simple and easy-to-use manner for constrained devices [12]. To the best of our knowledge, there is no standard representation for TS in IoT. Therefore, having a standard representation may be beneficial since it breaks the significant dependency between constrained devices and cloud applications, which makes IoT devices independent from the network or application, and helps to reduce the interoperability problems experienced in IoT networks.

## 4. Big Picture: The Proposed Architecture

**Figure 2:**
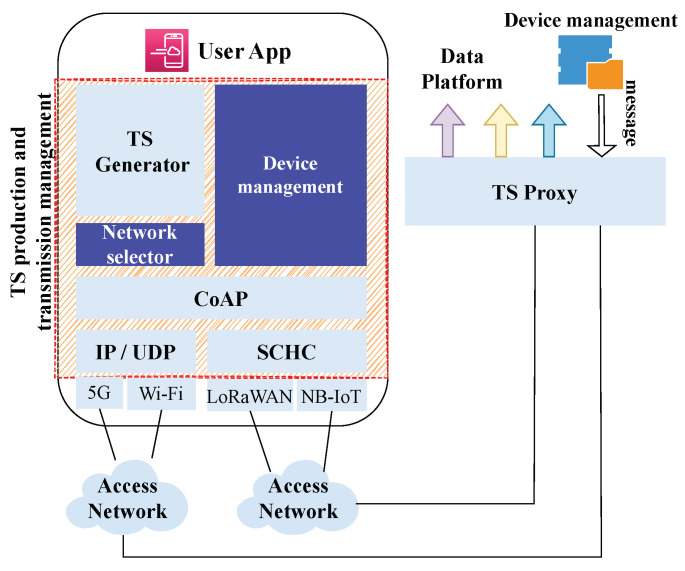
The proposed IoT architecture.

Figure 2 depicts our proposed architecture for IoT networks that encompasses the implementation of a middleware component between the data transmission process and the information processing process. The implementation of this middleware allows us to concentrate on the production of interoperable datasets. This, in turn, enables the seamless integration of IoT devices into various information systems without requiring any modifications to the actual code of the device. Then, our vision for the IoT architecture is centered on facilitating the integration of IoT devices into different information systems in an efficient and effective manner.

In our proposed architecture for IoT networks, the device user application is responsible for generating all the information obtained from the measured values. To facilitate the efficient management of the data transmission process and enable the seamless integration of IoT devices into various information systems, we propose to implement a middleware, placed between the transmission and information processing stages. Furthermore, the primary function of this part is to compactly group the data generated by a sensor using a common TS representation. This is a key contribution of our research. The middleware is also responsible for selecting the appropriate interface where the data will be transmitted, and for controlling the size and the periodicity of each measurement depending on the interface, band restrictions, and the type of measurement.

The transmission of data over wireless networks, such as Wi-Fi and 5G, requires the use of appropriate protocols to ensure efficient and reliable transmission. In this context, the use of the UDP/IP/CoAP protocol stack is commonly employed for the transmission of data to a proxy. To meet the specific requirements of networks such as LoRaWAN and NB-IoT, which have limited frame sizes, it is beneficial to employ techniques to reduce the size of the data being transmitted. Static Context Header Compression (SCHC) [37] is a header compression protocol, to reduce the size of the header information in the data packet. When compression is not enough, it also uses Fragmentation, to divide the data into smaller packets that can be transmitted more efficiently. Re-transmissions of lost packets is also provided to assure reliability. Thus, SCHC [37] could be used to compress IP/UDP/CoAP over LoRaWAN and NB-IoT which helps to meet the requirements of L2 word size [38,39]. (It must be noted, that the use of SCHC does not imply a full UDP/IP/CoAP protocol stack implementation, but since SCHC relies on compression rules, the device can process these rules directly to limit the footprint [40].)

Hence, the compact TS representation can be transmitted over various access networks such as 5G, Wi-Fi, LoRaWAN and NB-IoT, but ultimately all the data will converge at a TS proxy, as illustrated in Figure 2. The TS proxy, acting as an intermediary between the device and the data platform or cloud application, will be responsible for reconstructing the information transmitted by the device. Following the reconstruction process, the TS proxy will then proceed to convert the information from the TS standard representation into the specific data representation required by the data platform or cloud application. This conversion process is essential for ensuring compatibility and interoperability between the device and any data platform or cloud application.

## 5. CBOR Templates: An Optimization for TS Data Representation

The utilization of CBOR, a binary data format known for its extensibility, presents the opportunity to optimize the transmission process through the definition of new tags. As outlined in [41], the proposed use of a CBOR template allows for the handling of variables, thus enabling the transformation of data representation from formats such as JSON to CBOR. As defined by [41], a CBOR template is a CBOR data item that contains one or more variables. These variables are represented within the template as a CBOR data item that contains a specific identifier, commonly referred to as CBOR tag 42. This specific identifier allows for the efficient identification and handling of variables within the CBOR template, resulting in a reduction of the overall number of bytes transmitted during the process.

### 5.1. Static-based Time-Series (STS)

We present the STS proposition through the example present in Listing 11.

**Listing 11.** A template example present in [41].


 


  {

    ‘name":  ‘Carsten Bormann’,

    ‘place’: 42(0)

  }


 




where:

- 42: The Tag Number (TAGN), which indicates the variable identifier.
- (0): Indicates the position where the value of the variable is, in this case, the first position of a CBOR array.

It is important to note that, when the template outlined in Listing 11 undergoes the process of substitution, with the variable 0 assigned the value of “Bremen”, the resultant data item will be as depicted in Listing 12.

**Listing 12.** CBOR variable substituted in Listing 11 [41].


 


  {

    ‘name’:  ‘Carsten Bormann’,

    ‘place’: ‘Bremen’

  }


 




This example shows the limitation of the proposed template in [41] (i.e., Static-based Time-Series (STS)), which handles only static variables. Specifically, it is stated that when devices are required to transmit 100 values of the same variable, a separate variable must be utilized for each measurement, resulting in the need for 100 variables in the template. This limitation is exemplified in the context of the JSON structure presented in Listing 13, which pertains to a temperature and humidity sensor transmitting three simultaneous measurements.

**Listing 13.** A JSON structure that will be employed in the rest of the article.

[

    { ‘n’: ‘temperature’,  ‘u’: ‘Cel’, ‘v’: 32 },

    { ‘n’: ‘humidity’,   ‘u’: ‘%RH’, ‘v’: 20 },

    { ‘n’: ‘temperature’,  ‘u’: ‘Cel’, ‘v’: 31 },

    { ‘n’: ‘humidity’,   ‘u’: ‘%RH’, ‘v’: 21 },

    { ‘n’: ‘temperature’,  ‘u’: ‘Cel’, ‘v’: 30 },

    { ‘n’: ‘humidity’,   ‘u’: ‘%RH’, ‘v’: 22 }

]



Thus, the appropriate template as per the STS model would be present in Listing 14.

**Listing 14.** Example of Listing 13 as a template with CBOR variables.

[

    { ‘n’: ‘temperature’,  ‘u’: ‘Cel’, ‘v’: 42(0) },

    { ‘n’: ‘humidity’,   ‘u’: ‘%RH’, ‘v’: 42(1) },

    { ‘n’: ‘temperature’,  ‘u’: ‘Cel’, ‘v’: 42(2) },

    { ‘n’: ‘humidity’,   ‘u’: ‘%RH’, ‘v’: 42(3) },

    { ‘n’: ‘temperature’,  ‘u’: ‘Cel’, ‘v’: 42(4) },

    { ‘n’: ‘humidity’,   ‘u’: ‘%RH’, ‘v’: 42(5) }

]



The CBOR array that undergoes substitution in this scenario is shown in Listing 15.

**Listing 15.** CBOR array undergoinf the substitution in template from Listing 14.

[32, 20, 31, 21, 30, 22]



However, it should be noted that the STS model, as a static template, is unable to accommodate variations in the number of measurements in the data item. In the event that a different CBOR data item with a differing number of measurements is received (e.g., an array with only four measurements [32, 20, 31, 21]), the template would expect a different number of values (six variables) and would not be able to perform the substitution.

### 5.2. Tree Formatting

In this study, we present a novel approach for representing the location of variables in CBOR data items through the use of a tree structure within the TAGN model. This approach allows for a reduction in the amount of data transmitted by IoT devices and opens the possibility for the use of non-static templates in future developments.

As an illustration of this approach, we considered the example presented in Listing 16, which features nested arrays across four levels to demonstrate the tree representation. This representation allows for a clear and efficient identification of the location of variables within the CBOR data item.

**Listing 16.** Example with nested arrays to explain tree representation.


 


[

  [0, 1, 2, 3],

  [4, 5,

      [ 6, 7, 8, 9] ],

  [10, 11]

]



**Figure 3:**
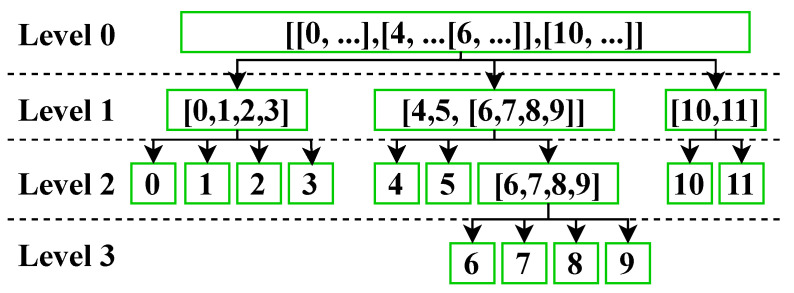
Tree representation in levels of Listing 16.

The tree representation of the array presented in Listing 16 is illustrated in Figure 3.

In the context of the proposed representation of the TAGN, it is feasible to selectively access specific values as needed. The following values can be accessed:

1. Consider the tag represented as TAGN (0,1), it refers to a specific value within an array of data. Specifically, it refers to the first array of data (TAGN (**0**,1)), in this case ([0,1,2,3]), and the second position of that array (TAGN (0,**1**)). Therefore, the value referred to by this tag is: **1**.
2. The tag represented as TAGN (1,2,3) refers to a specific value within an array of data. Specifically, it refers to the second array of data (TAGN (**1**, 2, 3)), in this case ([4,5[6,7,8,9]]), within that array, it refers to the third position (TAGN ( 1, **2**, 3)) which is another array ([6,7,8,9]), and finally the value present in the fourth position (TAGN ( 1, 2, **3**)) which is: **9**.
3. In addition to referring to specific values within an array of data, the proposed representation of the TAGN also allows for the inclusion of the value “true” which signifies the selection of all values present in an array. Consider the tag represented as TAGN (1, 2, true): It corresponds to all the values present in the array located at [1, 2]. This feature enables the efficient selection of multiple values within an array without the need to specify each individual position:
  (a) [ 1, 2, **0** ]: **6**
  (b) [ 1, 2, **1** ]: **7**
  (c) [ 1, 2, **2** ]: **8**
  (d) [ 1, 2, **3** ]: **9**

### 5.3. Delta between Measurements

In this section, we present the incorporation of delta values in CBOR templates as a means of reducing the amount of data transmitted by IoT devices. The utilization of deltas allows for the transmission of only the differences between subsequent measurements, rather than transmitting the entire measurement values.

To illustrate this concept, we provide the example presented in Listing 13. The proposed approach of using deltas is shown to effectively reduce the amount of data sent by IoT devices while preserving the integrity of the measurement data. Then, the CBOR data item for the STS template is necessary to reproduce Listing 13 is: [32, 20, 31, 21, 30, 22]. This data item requires 10 bytes when encoded using CBOR. By utilizing the delta values between subsequent measurements, the data size can be reduced. In this case, the difference in temperature is −1 (32 − 1 = 31, 31 − 1 = 30) and humidity is +1 (20 + 1 = 21, 21 + 1 = 22). Therefore, only the first value is necessary as a reference. As a result, the array using the delta between measurements is: [32, 20, −1, 1, −1, 1], which requires only 8 bytes rather than 10.

The utilization of deltas to represent measurements is particularly beneficial when the measurement values are higher. This is explained by the example of [535,537,539,538] which requires 13 bytes of data when encoded using CBOR. In contrast, the use of delta values [535,2,2,−1] reduces the data size to only 7 bytes. This first approach for delta uses the difference horizontally. However, it is possible to introduce a delta using the difference between the measurements vertically. Then, consider a CBOR template where the CBOR data item to replace is depicted by Listing 17.

**Listing 17.** Example of a CBOR data item. Four arrays with four values each one.


 


[   [535,537,539,538],

    [550,551,552,553],

    [572,574,573,570],

    [590,589,588,587]

]



Listing 17 requires 53 bytes when encoded using CBOR Then, when we use the horizontal delta, it transforms Listing 17 to Listing 18 using only 29 bytes instead of 53 bytes, reducing around 45% the number of bytes to represent the same information.

**Listing 18.** Example of a CBOR data item with horizontal delta representation. Each array contains the reference value (first position of the array), and then the difference between the subsequent values (i.e., 535+2=537, 537+2=539, 539−1=538).


 


[   [535,  2,  2, -1],

    [550,  1,  1,  1],

    [572,  2, -1, -3],

    [590, -1, -1, -1]

]



**Figure 4:**
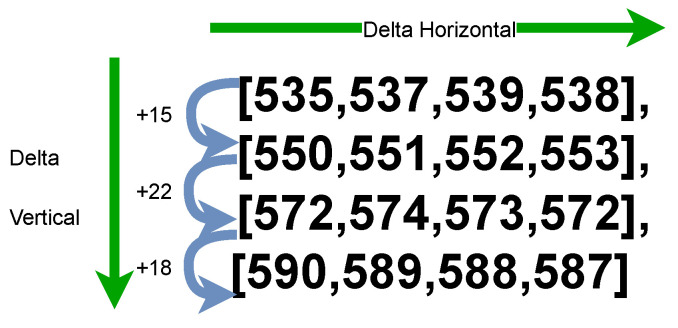
Representation of horizontal delta and vertical delta of Listing 18.

Moreover, the difference vertically (vertical delta) as depicted in Figure 4 is between each reference value (i.e., 535,550,572,590), thus with 535 as the reference value for all the measurements the difference will be (+15,+22,+18) respectively, (535+15=550,550+22=572,572+18=590) as shown in Listing 19.

**Listing 19.** Example of a CBOR data item with horizontal and vertical delta.


 


[   [535, 2,  2, -1],

    [15,  1,  1,  1],

    [22,  2, -1, -3],

    [18, -1, -1, -1]

]



The vertical delta is designed to be used when the data transmitted by the device corresponds to the same measurement when multiple sensor devices are used to collect the same type of information, e.g., humidity measurements. In this case, as the information does not change abruptly, it is possible to reduce the amount of data sent even more compared to only using the horizontal delta.

The utilization of delta values as proposed in this section results in a significant reduction in the amount of data transmitted by IoT devices. This is evidenced by the example presented in Listing 18, which requires only 23 bytes when encoded using CBOR. This represents a reduction of approximately 54% compared to when the delta is unused. As previously stated, in the subsequent sections of this study, all examples will be presented utilizing delta values (horizontally) as described in this section to further demonstrate the effectiveness of this approach in reducing data transmission size.

### 5.4. Variable-Based TS (VTS)

The utilization of CBOR variables and templates in Section 5.1 promises to reduce efficiently the amount of data transmitted by IoT devices. However, this approach is limited by the requirement of knowing the exact number of data items or variables to be replaced. This limitation can pose a challenge in certain cases, for example when the number of data items or variables is not known in advance.

In this section, we propose the inclusion of a new value to the TAGN specification. The incorporation of this new value addresses the limitation of the previous approach and makes it possible to represent TS data items that have an unknown number of data items.

This is achieved by using a template and an array of information depicted in Listing 20.

**Listing 20.** VTS template to represent Listing 13.

[

    { ‘n’: ‘temperature’, ‘u’: ‘Cel’, ‘v’: TAGN(0, true) },

    { ‘n’: ‘humidity’,   ‘u’: ‘%RH’, ‘v’: TAGN(1, true) }

]



It is important to note that the identification of the CBOR variables present in the CBOR data item can be achieved through the utilization of the TAGN tag. The remaining value in the TAGN specification indicates the location at which the corresponding value can be found within the data item. Then, the structure of the TAGN is represented in the format from Listing 21.

**Listing 21.** TAGN structure for VTS.


 


TAGN(v1, v2, .., vn)


 




Where:

1. TAGN is the tag number.
2. The values v1, v2, and vn indicate the locations where the respective variable values can be found:
  (a) If ‘true’, it indicates that all the items on the array should be selected.
  (b) If it is a number, it denotes the specific position or array where the value is.

In accordance with the structure of the template proposed, the CBOR data item required to represent the JSON structure presented in Listing 13 must be the one in Listing 22.

**Listing 22.** VTS CBOR data item to represent Listing 13 with VTS template present in Listing 20.


 


[ [32, -1, -1], [20, 1, 1] ].


 




It is important to note that the understanding of how the template works and how the CBOR data item was constructed is available in Section 5.2 and Section 5.3, respectively. Thus, the outcome of the template when values are replaced is depicted in Listing 23.

**Listing 23.** Final result for VTS with information replaced.

[

    { ‘n’: ‘temperature’,  ‘u’: ‘Cel’, ‘v’: 32 },

    { ‘n’: ‘humidity’,   ‘u’: ‘%RH’, ‘v’: 20 },

    { ‘n’: ‘temperature’,  ‘u’: ‘Cel’, ‘v’: 31 },

    { ‘n’: ‘humidity’,   ‘u’: ‘%RH’, ‘v’: 21 },

    { ‘n’: ‘temperature’,  ‘u’: ‘Cel’, ‘v’: 30 },

    { ‘n’: ‘humidity’,   ‘u’: ‘%RH’, ‘v’: 22 }

]



The utilization of the template presented in Listing 20 allows for a degree of flexibility in terms of the number of values that can be transmitted. This is due to the presence of the Boolean value ’true’ within the TAGN field.

As a result, it is possible to transmit multiple values for both humidity and temperature. As an example, the CBOR data item depicted in Listing 24 can be utilized to transmit two values for humidity and two values for temperature.

**Listing 24.** VTS CBOR data item with only two measurements.


 


[

    [32, -1],

    [20,  1]

]


 




Then, substituting the values from the CBOR data item in Listing 24 into the VTS template, it produces the output shown in Listing 25.

**Listing 25.** Result for VTS sending the two values from Listing 24.


 


[

    { ‘n’: ‘temperature’, ‘u’: ‘Cel’, ‘v’: 32 },

    { ‘n’: ‘humidity’,   ‘u’: ‘%RH’, ‘v’: 20 },

    { ‘n’: ‘temperature’, ‘u’: ‘Cel’, ‘v’: 31 },

    { ‘n’: ‘humidity’,   ‘u’: ‘%RH’, ‘v’: 21 }

]


 




**Figure 5:**
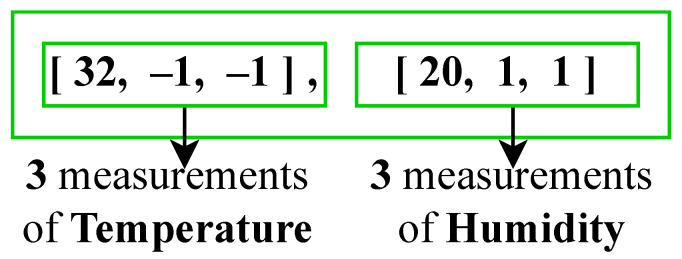
Explanation for the CBOR data item required in VTS.

In the last two examples, it is evident that there are two arrays present. These arrays are utilized to represent two distinct variables—temperature and humidity—independent of the number of measurements that are sent. Thus, this approach is organized by variables, as illustrated in Figure 5.

The use of arrays in this manner allows for efficient storage and manipulation of the data, as each variable can be accessed and processed separately. Furthermore, this approach ensures that the data remains organized and easily interpretable, even as the number of measurements increases or decreases. Overall, Variable-based TS (VTS) template provides a clear and structured way to handle large amounts of data in a meaningful way.

### 5.5. Metadata

Thus far, the examples presented have illustrated the usage of a basic payload. However, it is important to note that the data transmitted within the IoT context is often more complex and multifaceted. In addition to the actual measurement of the variable, certain use cases may require the inclusion of supplementary information, such as:

1. The timestamp of the measurement;
2. The identification of the sensor;
3. The precision of the sensor;
4. The unit of measurement for the sensor;
5. Among others.

To address the need for this additional information, we propose the incorporation of a feature referred to as ‘metadata’. This feature allows for the differentiation between the metadata and the measurements being transmitted.

Then, to facilitate the storage of this metadata, we propose the utilization of a map type in CBOR format. This map type will be present every time a message is sent, allowing for the clear and organized handling of the data sent by the IoT device. Thus, the metadata representation in the VTS template will be represented as in Listing 26.

Furthermore, as mentioned in Section 2.2, integers are optimized to be represented in CBOR. Thus, we use the integers 0,1,2,3,4 and 5 to represent the keys in the corresponding map, referring to the context, timestamps, precision, delta, and unit respectively.

**Listing 26.** Representation of metadata in VTS. The general metadata are in the first map; then, inside each array of measurements the specific metadata are present.


 


[  { General METADATA},

        [ {Specific METADATA}, 32, -1, -1 ],

        [ {Specific METADATA}, 20,  1,  1 ]

]


 




Finally, Table 2 introduces the metadata values defined in this proposal. Here, you can find a brief meaning, the value defined for each metadata value, the unit used to represent each metadata value, and an example, where the general or specific metadata can be found, and the default value when it is not present in the CBOR data item.

#### 5.5.1. General Metadata

General metadata are an essential element in the data representation proposed in this study. Thus, the values contained within the general metadata establish the context for all the values present in the CBOR data item. This context will ensure the accurate interpretation and utilization of the data.

- **1 and 2: Time Stamp**

      The time stamp is used to denote the point in time at which the measurements were taken. This is achieved by representing two values, the absolute time and the interval between each subsequent measurement:

  1. Time: Represented by the integer **1**. The value present in this key is represented using the CBOR tag 1 [16], which specifies the date and time in EPOCH format. As constrained devices have limited capabilities, normally the clock relays on the gateways or servers to provide the time information when needed [43]. Thus, if the time is not present in the metadata, the time of reception is used by default.
  2. Time difference: the time difference between successive measurements is indicated by the integer value **2**. This key gives information about the interval at which the measurements were taken. For instance, a value of 2 indicates that the measurements were taken every two seconds after the previous measurement. Conversely, a value of −5 would imply that the measurements were taken five seconds prior to the subsequent measurement. Finally, the time between measurements for IoT applications depends on several factors e.g., the network bandwidth, sensor sampling rate, etc [44]. Then, we have selected 60 s as the default value when no value is provided in the metadata. This is a medium value that is neither too high for applications that send data every few minutes nor too low for applications that send data every few seconds.
     Listing 27 presents an example of a time stamp present in the metadata.

**Listing 27.** Example of a time stamp in the metadata. The number 1 represents the time and the corresponding value is in EPOCH format, while the number 2 represents the difference in seconds between measurements, in this case, corresponding to 30 s.

 

[   { 1: 1654070400,

     2: 30 }  ]

      Listing 27 depicts that the initial measurement in each array of the CBOR data item was recorded on **Wednesday, 1 June 2022 at 10:00:00**. The subsequent measurements were acquired with an interval of **30** s. This would result in the second measurement being recorded on Wednesday, 1 June 2022 10:00:30, the third measurement on Wednesday, 1 June 2022 10:01:00, and so forth.

- **3: Precision**

      Precision refers to the decimal component of a floating-point number. For example, if we were to consider the numbers 2.345 and 2.34, both of which are classified as floating-point numbers, we would see that the former has a precision of 3, while the latter has a precision of 2.
      It is also noteworthy that floating-point numbers are not optimized in CBOR, as an integer value would be deemed more favorable due to its ability to represent a value with a smaller number of bytes. Therefore, if the precision can be indicated, the decoder will be able to interpret and accurately represent the values in question. Consequently, if the number 2.345 were to be represented, it would be represented as the integer 2345, with a precision of 3. In the event that the precision is included in the general metadata, all variables will possess the same precision. However, if precision is not present in the metadata, the default value will be 2. The precision of IoT data may vary depending on several factors, such as the sensor type, the data processing method, the environment, and others. Therefore, 2 is a suitable value that can meet this requirement for many applications.
      Thus, precision is represented by the integer **3** and an example of a precision present in the metadata is shown in Listing 28.

**Listing 28.** Example of a Precision value in the metadata. The value one refers to only one decimal position for precision.


 



[


    { 3: 1 }


]

      Listing 28 represents that the measurements present in the CBOR data item have a precision of 1. Then, if a measurement inside the CBOR data item has a value of 145 the final value would be 14,5.

- **4: Delta**

      The difference between consecutive values, referred to as delta, is described in Section 5.3 and can be either horizontal or vertical in nature.
      The presence of delta is indicated by the integer **4**. The value within the CBOR metadata object represents the orientation of delta, with 1 indicating a solely horizontal orientation, 2 indicating both horizontal and vertical, and 10 indicating delta being inactive. In the absence of delta being specified in the metadata, a default value of 1 is assumed. In the same direction as other works such as [45], delta encoding typically involves only horizontal differences. An example of a delta value as found within the metadata is presented in Listing 29.

**Listing 29.** Example of a delta value in the metadata. The value 10 corresponds to the delta being inactivated.

 

[

    { 4: 10 }

]

      Thus, Listing 29 shows that the measurements do not have to be managed as the difference of the subsequent values.

- **0: Context**

      Context is mandatory to be always present in the general metadata, and can refer to information such as the precision of measurements, the time difference between each measurement, and many other relevant details.
      In the proposed data representation, context is represented by the integer **0**. The associated value to this key is an unsigned integer and represents the identification of the context for all the metadata within the system. For example, if we send the data {0:1}, we are referring to context **1** for all metadata. This context may include information such as the precision of measurements, the time difference between each measurement, and other supplementary information. It is important to note that the context can change by sending another value associated with the key 0, e.g., {0:2}. This allows the system to be flexible and the context to be easily updated as required.
      An important thing to remember is that once the context has been sent and the end device knows the corresponding value, it is only necessary to send the context in all the metadata. To explain this, consider the example in Listing 30.

**Listing 30.** Example of context value in the metadata. The context 1 refers to the difference in time (30 s) and the precision for the measurements (3).

 

[

    {  0: 1,

      1: 1654070400,

      2: 30,

      3: 3

    }

]

      Thus, the Listing 30, when represented in CBOR, takes 15 bytes. Then the context (number 1) shows that all the measurements were taken on **Wednesday, 1 June 2022 at 10:00:00** (1:1,654,070,400), and the subsequent measurements were taken **30** s after (2: 30). Finally, all measurements have a precision of **3** (3: 3) and, as the delta is not specified, it is set to **horizontal** by default. Furthermore, if the end device knows the context (1), the succeeding CBOR data items will only contain the metadata present in Listing 31.

**Listing 31.** General metadata when the context is known. The context is referring to the values sent in Listing 30.

 

[

    { 0: 1 }

]

      Finally, the metadata in Listing 31 is only 4 bytes when represented in CBOR instead of 15 bytes as in Listing 30.

#### 5.5.2. Specific Metadata

Specific metadata can be found in the measurement array, as shown in Listing 26. This specific metadata have the ability to override the general metadata, but if not present, the general metadata will apply. Furthermore, the values specified in Section 5.5.1 can be altered except for the context. Then, consider Listing 32 as an example of specific metadata.

**Listing 32.** Example of specific metadata. The values 0,1,3 as general metadata are replaced by the values 2:45 and 3:4 in the specific metadata.


 


[

    { 0: 1, 1: 1654070400, 2: 30, 3: 3 },

    [ { 2: 45} ],

    [ { 3: 4} ]

]



      Listing 32 gives an example where the specific metadata replaces the general metadata on two separate occasions:

1. In the first array of measurements, it overwrites the time difference (2). Now, the measurements are not taken every 30 s, instead, the value indicates that each measurement inside the array is taken every 45 s.
2. In the second array of measurements, it overrides the precision (3). Now, the measurements no longer have a precision of 3, instead, the value indicates that each measurement now has a precision of 4.

- **5: Unit**

      The unit, represented by the integer **5**, identifies the metric unit used for each variable taken. To ensure validity, all possible values for this key must be listed in [42]. Thus, when the value does not exist in this list, it will not be accepted by the VTS template. As an example, Listing 32 can be modified to reflect that the first array of measurements is for a temperature sensor measuring in degrees Celsius. The result of this modification is shown in Listing 33.

**Listing 33.** Example of specific metadata with unit specification. In this case, the value 5: ‘Cel’ in the object where is specified, the measurements are in Celsius degrees.

 

[

    { 0: 1, 1: 1654070400, 2: 30, 3: 3 },

    [ { 2: 45, 5: ‘Cel’} ],

    [ { 3: 4} ]

]



### 5.6. CDDL Specification

As outlined in Section 2.5, Concise Data Definition Language (CDDL) enables the definition of data structures that are both machine-processable and human-readable. Then, the objective of this section is to provide the CDDL code that outlines the data structure proposed in this article through the use of the VTS template and metadata.

Listing 34 defines the array for the VTS template, composed by a template-context and the data values of the measurements present in the template.

**Listing 34.** VTS array represented in CDDL code. The data format is defined by a context and data-values.


 


; Definition of general array

template-array-vts = [

                          template-context, ; General Metadata

                        + data-values, ; Data values

                    ]



Once the general array is defined, it is necessary to declare the template-context and the data-values. Therefore, Listing 35 illustrates the template-context, which consists of the Context (0), present in Section 5.5.1, and all other values found in the general metadata such as time, precision, and delta.

**Listing 35.** Template-context definition in CDDL code. Definition of general metadata and the corresponding values.


 


; Definition of template context

template-context = {

        0 : uint, ; Context per message

      ? general_metadata

    }


 


; Definition of general metadata

general_metadata = (

      0*1 1 : epoch, ; Epoch Time

      0*1 2 : uint, ; Difference in time in seconds

      0*1 3 : precision, ; Precision

      0*1 4 : delta ; Difference with the subsequent value

)



Finally, Listing 36 represents the arrays for the data values, composed by the specific metadata, depicted in Section 5.5.2, and the integers values from the measurements.

**Listing 36.** Specific metadata and data values definition in CDDL code.


 


; Definition of specific metadata

specific_metadata = (

      general_metadata,

      0*1 5 : unit ; Change of unit

)


 


; Definition of Data Values (Measurements)

data-values  = [

      0*1 {specific_metadata},

      + values: int

]



Listings 34–36 depict a subset of all the proposed rules in the proposed data format. Once the proposed data format is defined with CDDL it is possible to validate that any CBOR data structure is well structured, or to create an example of the data structure with random values. Listing 37 presents the command to generate a random example with the structure defined in a file named CBOR_Template_VTS.cddl, and described in [46], with the corresponding result.

**Listing 37.** CDDL command to generate a random example and the result. The random example follow all the structure defined in the code from Listings 34–36 saved in the archive CBOR_Template_VTS.cddl.


 


>> cddl CBOR_Template_VTS.cddl generate 1


 


[{0: 3622, 3: 7, 4: 10}, [1180, -91, 1771, 756],

[{3: 3, 4: 10}, 2849, -809, 3896], [2732, -690]]


 




Finally, a full description and explanation of how to use the CDDL code described in this section is presented in [46].

## 6. Performance Evaluation

In this section, we present a comprehensive performance evaluation of the proposed time series representation format VTS comparing against JSON, and other binary formats as CBOR, Protocol Buffers (protobuf) and ASN.1. The evaluation is divided into five parts: Payload Comparison, Fragmentation, Time-on-Air, Battery Lifetime, and Metadata Comparison.

In the Payload Comparison, we compared the payload of the proposed representation format against the conventional JSON representation and binary formats as CBOR, Protocol Buffers (protobuf) and ASN.1. The Fragmentation section evaluated the impact of the proposed representation format on the packet Fragmentation when the payload is sent over a LoRaWAN network. Consequently, in the Time-on-Air section in a LoRaWAN network, we calculated the time required to transmit the data collected by IoT devices using VTS, JSON, Protocol Buffers (protobuf), ASN.1, and CBOR representation formats. Then, in the Battery Lifetime section, we evaluated the impact of the proposed representation format on the Battery Lifetime of IoT devices. Finally, we compared the metadata proposed in this article with the metadata proposed in [17].

### 6.1. Payload Comparison

In the context of IoT devices, payload size is a critical factor that affects the performance and efficiency of wireless communication. A larger payload size means more Time-on-Air, which consumes more energy and reduces the battery life of IoT devices. Moreover, a larger payload size also increases the risk of packet loss and Fragmentation due to channel interference and noise. Therefore, in this section, we compared two payloads: the payload used throughout this article as depicted in Listing 13 and the payload from real world data based on GPS measurements.

#### 6.1.1. Article Example

Consider the JSON representation example depicted in Listing 38.

**Listing 38.** The JSON representation of Listing 13 to be transformed into CBOR, and VTS.

[ {‘n’:‘temperature’, ‘u’:‘Cel’, ‘v’:32},

  {‘n’:‘humidity’,  ‘u’:‘%RH’, ‘v’:20},

  {‘n’:‘temperature’, ‘u’:‘Cel’, ‘v’:31},

  {‘n’:‘humidity’,  ‘u’:‘%RH’, ‘v’:21},

  {‘n’:‘temperature’, ‘u’:‘Cel’, ‘v’:30},

  {‘n’:‘humidity’,  ‘u’:‘%RH’, ‘v’:22} ]



**Figure 6:**
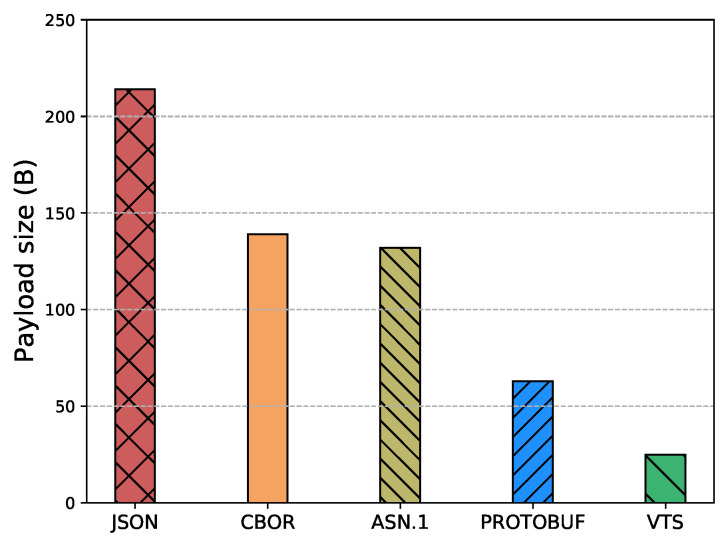
Payload size comparison between JSON, CBOR, ASN.1, Protocol buffers (Protobuf) and VTS template.

The payload size required to transmit all the data in this JSON representation is 214 bytes. In contrast, the payload size required for the CBOR representation is 139 bytes, while the payload size to represent it in Protocol buffers and ASN.1 is 132 and 63, respectively. However, the VTS template requires a significantly smaller payload size of 25 bytes. Then, as depicted in Figure 6, the implementation of the VTS proposal allows for a substantial reduction of approximately 88% in payload size (from 214 bytes to 25 bytes). Moreover, compared to Protocol buffers, the VTS representation reduces approximately 60% in payload size (from 63 bytes to 25 bytes)

#### 6.1.2. Real World Data

To test the template proposal using real world data, we used the dataset 11 from the repository [46]. This dataset consists of the first 300 observations obtained from a GPS placed on a bicycle to promote cyclist safety in smart cities [47]. The GPS system includes latitude, longitude, and velocity information, resulting in a total of 900 measurements.

Listing 39 presents the JSON format with the SenML structure that must be employed for data transmission. In this instance, it necessitates 42,843 bytes for successful transmission.

**Listing 39.** The JSON representation of the GPS real world data.


 


[ {’n’:’Latitude’, ’u’:’Lat’,’t’:1667135896307 ,’v’:-29.620745},

 {’n’:’Longitude’, ’u’:’Lon’,’t’:1,’v’:-51.127167},

 {’n’:’Velocity’, ’u’:’m/s’,’t’:1,’v’:0.024192}

                  .

                  .

                  .

 {’n’:’Latitude’, ’u’:’Lat’,’t’:1,’v’:-29.622693},

 {’n’:’Longitude’, ’u’:’Lon’,’t’:1,’v’:-51.131297},

 {’n’:’Velocity’, ’u’:’m/s’,’t’:1,’v’:12.534}    ]



**Figure 7:**
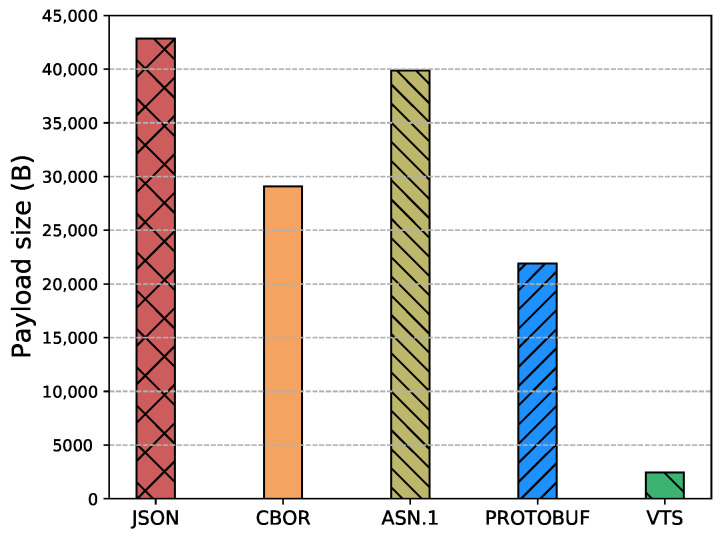
Payload in bytes comparison between JSON, CBOR, ASN.1, Protocol buffers (protobuf) and VTS template for real world data.

Finally, as illustrated in Figure 7, while the implementation of CBOR reduced the payload size around 32% (from 42,843 bytes to 29,100 bytes), the use of the VTS template facilitated a decrease of nearly 94% in payload size (from 42,843 bytes to 2437 bytes). Even compared to Protocol buffers, which reduce by around 49% when compared to CBOR, the VTS proposal reduces the payload size 88% (from 21,900 bytes in Protocol buffers to 2437 bytes).

### 6.2. Fragmentation

The Payload Comparison evaluation presented in Section 6.1 can be further extended to include payload Fragmentation during transmission over LoRaWAN. The Table 3 shows the maximum payload size for LoRaWAN within the EU 863–870 MHz band, depending on each Spreading Factor (SF) [48].

Determining the maximum payload size provides the necessary information to evaluate whether Fragmentation will occur during the transmission of each payload over LoRaWAN for each SF. This evaluation was achieved by using Equation (1).

(1) $$Number\;of\;fragments=\frac{payload}{Max\;payload\;per\;SF}.$$

**Figure 8:**
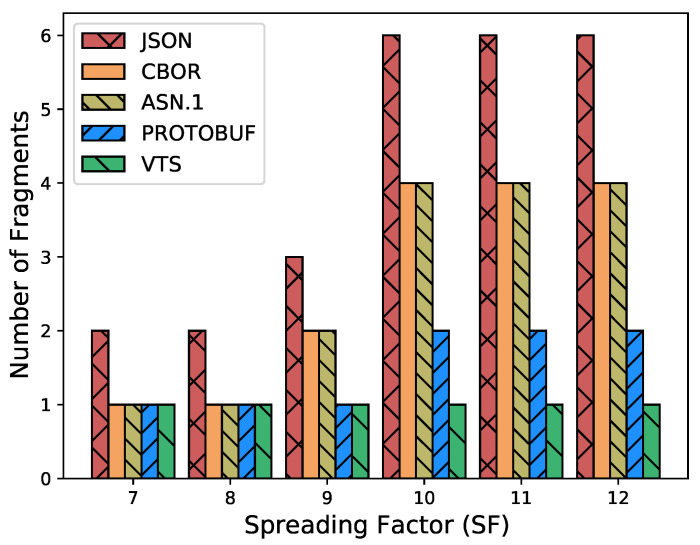
Fragmentation for the article example depicted in Listing 13 per SF.

**Figure 9:**
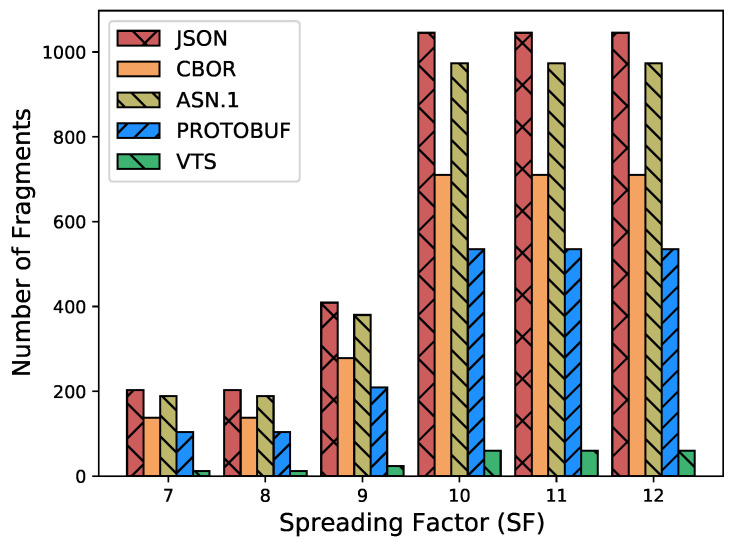
Fragmentation for GPS real world data per SF.

The evaluation outcome is presented in Figure 8 and Figure 9.

Furthermore, it is of paramount importance to incorporate the protocols, in this case, IP/UDP and CoAP, for the purpose of transmitting the payload information, accompanied by the necessary headers essential for LoRaWAN transmission. Thus, without SCHC the IP/UDP and CoAP headers, we need a total of 32 bytes. However, due to the implementation of the SCHC compression mechanism, the adoption of an appropriate rule to represent the IP/UDP headers significantly reduces this requirement to only 1 byte. Consequently, the CoAP headers necessitate an allocation of 4 bytes. Finally, the LoRaWAN headers demand a total of 13 bytes, encompassing 5 bytes in the MAC layer and 8 bytes in the application layer. Hence, to enable a comprehensive payload Fragmentation comparison, it becomes imperative to include an aggregate addition of 18 bytes per fragment.

As depicted in Figure 8, it shows that the payload in Listing 38, when using JSON format, a size of 214 bytes, will be fragmented into 6 fragments when using SF 10, 11, and 12, 2 fragments when using SF 7 and 8, and 3 fragments when using SF 9. A similar situation occurs when a payload of 139 and 132 bytes is represented in CBOR and ASN.1, respectively, it will be fragmented into 2 and 4 fragments depending on the Spreading Factor. Then, Protocol buffers are only fragmented when using SF 10, 11, and 12 into 2 fragments. However, a payload of 25 bytes length, if represented in VTS format, will not be fragmented at any Spreading Factor.

Finally, as illustrated in Figure 9, the payload in Listing 39 when using VTS format, with a size of 2437 bytes, will be fragmented between 12 and 60 fragments. On the other hand, the CBOR, ASN.1, Protocol buffers and JSON representations are expected to result in a Fragmentation between 104 and 1045 fragments.

### 6.3. Time-on-Air

An additional method to assess our proposal is through the examination of the Time-on-Air for the associated payload. The determination of Time-on-Air will facilitate in the next section the calculation of the battery life of an IoT device utilizing LoRa to transmit the data. To determine the time required to transmit a message over LoRa, the procedures, the modulation specifics, and the associated regional parameters for LoRa are taken into account [48,49,50].

The transmission time, denoted as $T_{packet}$, can be expressed as the summation of the preamble transmission time and the payload message transmission time, as depicted in Equation (2) [49].

(2) $$T_{packet}=T_{preamble}+T_{payload}.$$

Then, $T_{preamble}$ can be obtained with Equation (3) [49].

(3) $$T_{preamble}=\left(n_{preamble}+4.25\right)\times T_{symbol},$$

where $T_{symbol}$, presented in Equation (4), and $T_{payload}$, presented in Equation (5), can be defined as in [49].

(4) $$T_{symbol}=\frac{2^{SF}}{BW}$$

(5) $$T_{payload}=PLSymb\times T_{symbol}.$$

Finally, the number of symbols transmitted as the physical message without preamble is represented as PLSymb [50] in Equation (6).

(6) $$PLSymb=8+max\left(ceil\left(\frac{8\cdot PL-4\cdot SF+28+16\cdot CRC-20\cdot H}{4\cdot (SF-2\cdot DE)}\right)\cdot \left(CR+4\right),0\right),$$

where [49]:

- PL: Total bytes from the payload.
- SF: Spreading Factor.
- *H*: 1 if the header is not used otherwise 0.
- DE: Low Data Rate Optimization, 1 if used otherwise 0.
- CR: Code Rate, from 1 to 4.
- BW: Bandwidth.

Thus, with EU863-870 MHz Band, a bandwidth of 125 MHz (BW =125,000), a code rate of 1 (CR=1), header disabled (H=0), and not using low data rate optimization (DE=0) [49,50], we proceeded to calculate the Time-on-Air for all the payloads from Section 6.1 (214 bytes, 139 bytes, 132 bytes, 63 bytes, 25 bytes, 42,843 bytes, 29,100 bytes, 39,878 bytes, 21,900 bytes, and 2437 bytes).

**Figure 10:**
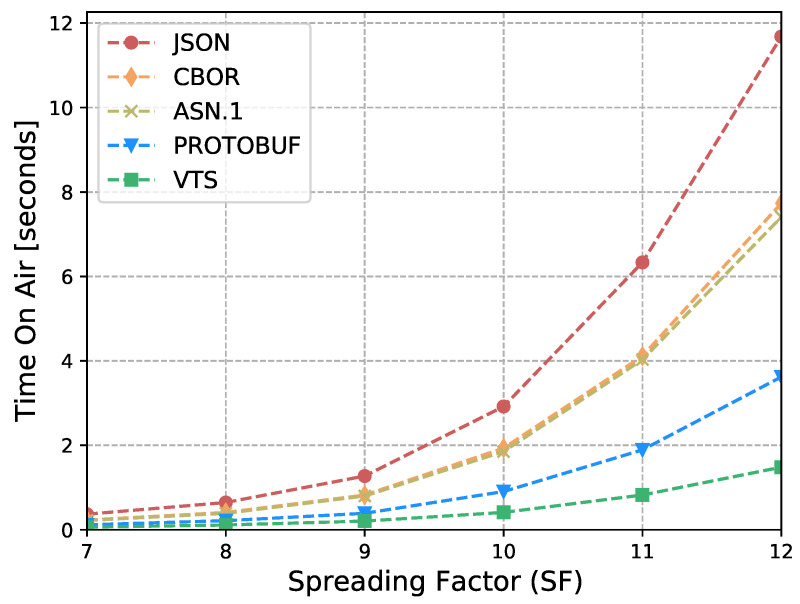
Time-on-Air comparison for article payload example in Section 6.1.1 per SF.

Figure 10 shows that, for Listing 13 when using the VTS format, a payload with a size of 214 bytes will be transmitted between 0.06 s and 1.48 s, depending on the SF. When using JSON, CBOR, ASN.1 and the Protocol buffers format, as the payload increases, it will be transmitted between 0.37 s and 11.68 s for JSON, between 0.23 s and 7.73 s for CBOR, between 0.22 s and 7.40 s for ASN.1 and between 0.11 s and 3.62 s for Protocol buffers. Thus, the VTS reduces by around 87% (from 11.68 s to 1.48 s) when compared to JSON, 81% (from 7.73 s to 1.48 s) when compared to CBOR, 80% (from 7.40 s to 1.48 s) when compared to ASN.1, and 59% (from 3.62 s to 1.48 s) when compared to Protocol buffers in SF 12.

**Figure 11:**
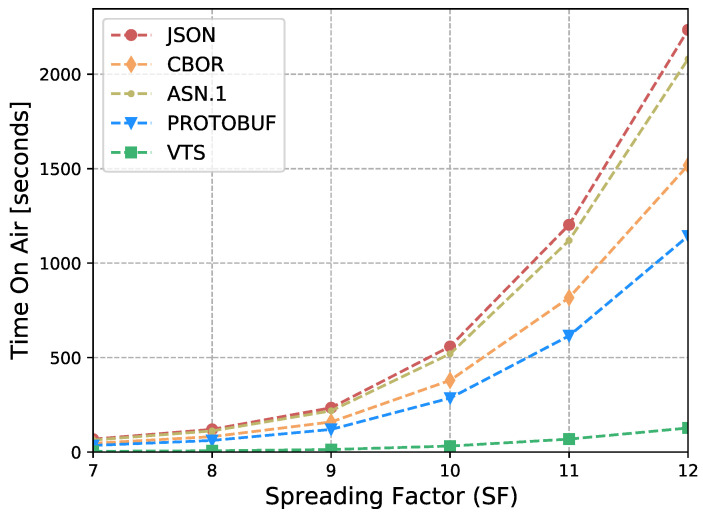
Time-on-Air comparison for real-world data payload in Section 6.1.2 per SF.

Figure 11 shows that, for Listing 39 when using the VTS format, reduces by around 94% (from 2234 s to 127 s) when compared to JSON, 91% (from 1517 s to 127 s) when compared to CBOR, 93% (from 2079 s to 127 s) when compared to ASN.1, and 88% (from 1142 s to 127 s) when compared to Protocol buffers in SF 12.

Additionally, when comparing Figure 8, and Figure 9 demonstrates that the SF has a direct effect on the LoRaWAN Time-on-Air. However, an increase in the SF results in an increase in the Time-on-Air. Then, the magnitude of this impact can be reduced by reducing the size of the payload which is the achievement when the VTS template is utilized.

### 6.4. Battery Lifetime

One of the key evaluations to determine the impact of the VTS template implementation, is the comparison of the battery life of an IoT sensor device in relation to the payload. Thus, a simple linear function has been employed to calculate the Battery Lifetime [50]. This function considers the battery capacity ($C_{battery}$ in mAh) and the average current consumption ($I_{Avg}$ in mA) of the IoT device as the key parameters in Equation 7 [50].

(7) $$T_{lifetime}=\frac{C_{battery}}{I_{Avg}}.$$

As depicted in Equation 8, in order to determine the average current consumption of an IoT device, several factors must be considered:

- The total time between two consecutive packet transmissions ($T_{app}$ in seconds).
- The number of states ($N_{states}$) through the IoT device goes through to transmit a packet.
- The current consumption ($I_{i}$ in mA) of each state for the IoT device.
- The time duration ($T_{i}$ in seconds) of each state for the IoT device [50].

(8) $$I_{avg}=\frac{1}{T_{app}}\cdot \sum_{i=1}^{N_{states}}I_{i}\cdot T_{i}.$$

Thus, considering an IoT device, which implements four states to transmit a packet—sleep (Sleep), stand by (Stb), reception (Rx), and transmission (Tx) [51]:

(9) $$I_{avg}=\frac{1}{T_{app}}\cdot \left(I_{Rx}\cdot T_{Rx}+I_{Tx}\cdot T_{Tx}+I_{Sleep}\cdot T_{Sleep}+I_{Stb}\cdot T_{Stb}\right).$$

Consequently, Table 4 depicts the current in mA per state; in this case, we considered the transceiver Semtech SX1272 [52].

The values listed in Table 4 and the transmission time (Time-on-Air) as obtained in Section 6.3 are known. Then, to calculate the Battery Lifetime of an IoT device, it is necessary to determine the time in reception, standby, and sleep modes. As the SX1272 transceiver is a LoRaWAN Class A device [52], the reception time can be computed using Equation (10).

(10) $$T_{Rx}=\frac{2^{SF}}{N_{RxSymbols}}\cdot +\frac{2^{SF}+32}{BW},$$

where $N_{RxSymbols}$ is 10 for SF 11 and 12, and 8 for SF 7, 8, 9 and 10 [50].

Stand by time is generally 1.5 μs [50]. Consequently, sleep time, presented in Equation (11), will be the rest of the time when the application is not in any other state as transmission, reception or stand by.

(11) $$T_{Sleep}=T_{App}-T_{Tx}-T_{Rx}-T_{Stb}.$$

Finally, we considered two different application scenarios for the payloads present in Section 6.1.1 and Section 6.1.2. For Section 6.1.1, an application with a battery capacity of ($C_{battery}=2700$ mAh) and transmitting four packets per hour is presented. Furthermore, Section 6.1.2 presents an application with a battery capacity of ($C_{battery}=3700$ mAh) and transmitting one packet per hour.

**Figure 12:**
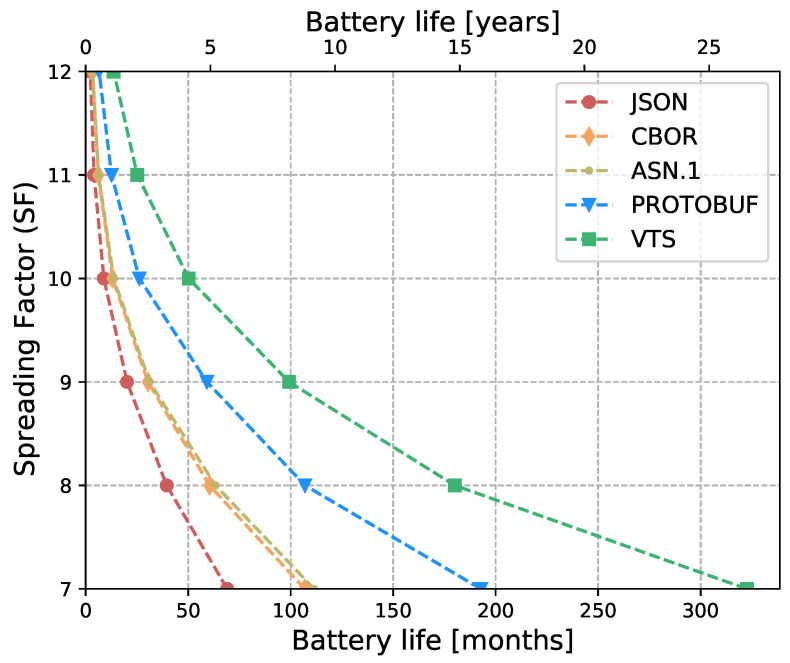
Battery lifetime comparison for payloads in Section 6.1.1 per SF.

Thus, Figure 12 depicts the increase of the battery life thanks to the use of VTS template. It shows an increase of almost 4.6 times in all of the SF (e.g., from 5.67 years to 26.51 years in SF 7) when compared to JSON. Moreover, an increase of 3.02 times compared to CBOR, an increase of 2.89 times compared to ASN.1, and an increase of 1.67 times compared to Protocol buffers.

**Figure 13:**
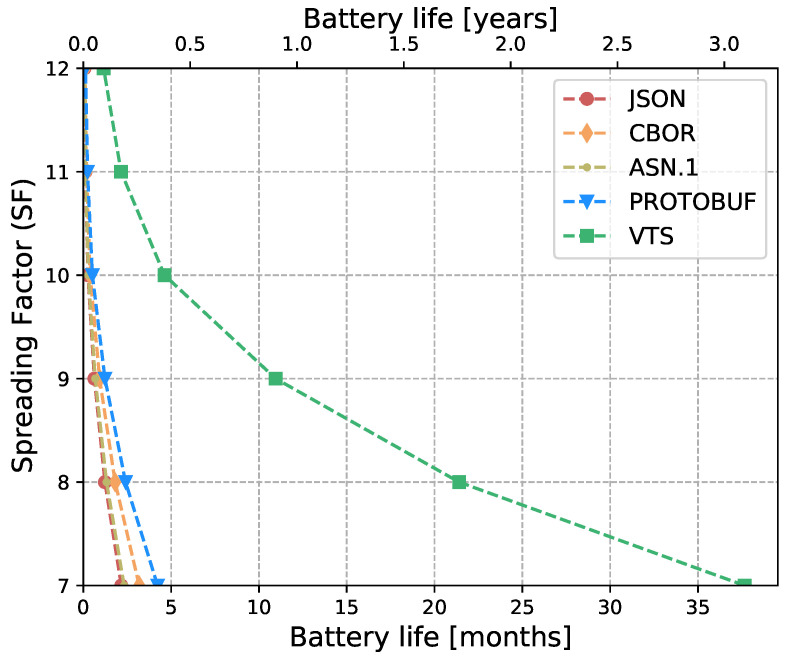
Battery lifetime comparison for payloads in Section 6.1.2 per SF.

Consequently, Figure 13 shows a nearly 17-fold increase in battery life in all SF (e.g., from 1.23 months to 21.4 months in SF 8) compared to JSON, a nearly 12-fold increase compared to CBOR, a 16-fold increase compared to ASN.1, and a nearly 9-fold increase compared to Protocol buffers.

Finally, the selected model for estimating battery life in IoT devices adopts a simplified approach, aiming to provide a quick estimation of battery life. It focuses on fundamental parameters such as power consumption, battery capacity, and average current consumption. However, it is important to note that this model does not incorporate certain influential factors such as the distance from the base station, link budget, re-transmissions, aging of the battery, or collisions. While the simplicity of the model can be beneficial in terms of ease of use, it is advisable to consider these additional factors for a more comprehensive and accurate estimation of battery life in practical scenarios.

### 6.5. Metadata Comparison

In this section, we evaluated the impact of the new metadata proposed in this article against the metadata proposed in [17]. Consider the metadata defined in Listing 40. This metadata corresponds to a payload of 9294 bytes in the dataset 3 from [46].

**Listing 40.** Old definition example of Metadata in [17].


 


{bt: 1593982800, dt:10, bp:3}



The first thing to note is that the proposed metadata takes into account that strings are not optimised in CBOR, and therefore, strings such as “bp” are represented as the integer 3.

Another aspect is the use of the “context” in the metadata proposed in this article. In comparison, the metadata outlined in [17] requires the transmission of all metadata in every packet. In this proposal, once the proxy has gained knowledge of the “context”, it is no longer necessary to transmit the full payload, but only the “context” is transmitted.

The representation of the metadata in Listing 40 in the metadata defined in Section 5.5 is depicted in Listing 41. It consists of 13 bytes as opposed to the 17 bytes used in Listing 40. Furthermore, when only the “context” 0:1 is transmitted, it is represented by only 3 bytes in CBOR.

**Listing 41.** New definition example of metadata in [17].

{0:1, 1: 1593982800, 2:10, 3:3}



Table 5 compares the total number of bytes required to represent metadata. It also shows the percentage of the metadata size in bytes compared to the payload represented in 9294 bytes. The analysis was conducted considering three cases: the first case represents a LoRaWAN network operating with a SF of 7 and the maximum allowed payload size, the second case represents a Wi-Fi network with a Maximum Transmission Unit (MTU) of 1468 bytes, and the third case depicts an LTE/5G network with an MTU of 1428 bytes [17].

In addition to the size reduction in the payload, it is also important to note the flexibility of the metadata thanks to the specific metadata depicted in Section 5.5.2. In addition to the reduction in payload size compared to [17] for the metadata, it also offers flexibility thanks to the specific metadata presented in Section 5.5.2. For example, Listing 42 allows the specification of different measurement periodicity, such as temperature readings every 30 s and humidity readings every 45 s, which is not possible in the metadata defined in [17].

**Listing 42.** Change of periodicity for different measurements in metadata. The key-value 2:value for each map that can be identified overrides the periodicity in the general metadata.

[{0:2, 1: 1593982800},[{2:30,5:‘Cel’},],[{2:45,5:‘%RH’},]]



Moreover, as in Listing 42 is also possible to change the unit of the measurement 5:‘Cel’ which is not currently possible in the metadata described in [17].

## 7. Discussion

In this section, we delve into certain use cases that may arise during the transmission of data. Our discussion aims to explore whether it is possible to represent the measurements in alternative metric units. Additionally, we explore what happens if a package containing the context of the measurements is lost. Moreover, we analyze scenarios where time series representation is not necessary in the network due to the real-time nature of the data. Lastly, we examine how it is feasible to transform data into other formats apart from the commonly used SenML or JSON. These use cases will help us in assessing the robustness and flexibility of the proposed approach, especially in the face of unforeseen challenges and issues that may arise in real-world IoT applications.

### 7.1. Other Units

One aspect that requires revision is the units of measurement defined in the metadata. Even though they are standardized by the IANA, there are certain units that may not be in this definition. For example, when utilized for GPS, the units latitude and longitude are defined, but degrees (°), minutes (’), and seconds (") (DMS coordinates) are also possible to use to represent GPS coordinates. However, this representation is difficult to represent using our definitions for VTS template and metadata, and may not reduce the amount of data transmitted. For instance, consider Listing 43 a coordinate example in DMS format, this uses 13 bytes to be represented in JSON format, while in CBOR it uses 15 bytes.

**Listing 43.** GPS coordinate example in DMS format. 

[

40° 51’ 59’’ N

]



To be represented using our data format and the VTS template, it would be necessary to translate the DMS format to decimal format and then it will be possible to transmit the corresponding information, which implies a double translation process leading to an increase in the processing power and time used to translate the data.

### 7.2. Lost Context

Another aspect that requires consideration is the context defined in the metadata. It can decrease considerably the amount of data needed to represent the metadata in different measurements; however, if a message is lost during a specification of the context, then default values will be taken when translating the data. This may result in errors in the final representation or even a lack of knowledge of the unit of each measurement present in the time series. For example, consider the GPS data represented in Listing 44.

**Listing 44.** The JSON representation of the first six measurements in the GPS real world data.

[  {’n’:’Latitude’,’u’:’Lat’,’t’:1667135896307 ,’v’:-29.620745},

  {’n’:’Longitude’,’u’:’Lon’,’t’:1,’v’:-51.127167},

  {’n’:’Velocity’,’u’:’m/s’,’t’:1,’v’:0.024192}

  {’n’:’Latitude’,’u’:’Lat’,’t’:1,’v’:-29.622693},

  {’n’:’Longitude’,’u’:’Lon’,’t’:1,’v’:-51.131297},

  {’n’:’Velocity’,’u’:’m/s’,’t’:1,’v’:12.534}

]



Then, the corresponding VTS template is depicted in Listing 45.

**Listing 45.** VTS template for Listing 44.

[  {’n’:’Latitude’,’u’:’Lat’, ’v’:TAGN(0,True)},

  {’n’:’Longitude’,’u’:’Lon’,’v’:TAGN(1,True)},

  {’n’:’Velocity’,’u’:’m/s’,’v’:TAGN(2,True)}

]



After the data processing to build the context and the CBOR data item to send the first two measurements of the GPS data are depicted in Listing 46 and Listing 47 respectively.

**Listing 46.** Context for CBOR data item needed to send the first three measurements of the GPS data. 

[ {0:1, 1:1667135896307, 2:1, 3:6} ]



**Listing 47.** CBOR data item needed to send the first three measurements of the GPS data. 

[

    {0:1},

    [-29620745,-1984],

    [-51127167,-4130],

    [24192,11658]

]



In case the context is lost, the translation into the JSON data format presented in Listing 44 will face errors due to it will not have a context, and will select the default values (precision of 2 instead of 6, the reception time will be taken instead of 1667135896307, and a difference in time of 60 s instead of 1 s), which differs from reality leading a wrong representation depicted in Listing 48.

**Listing 48.** The JSON representation of the GPS real world data with context lost.

[ {’n’:’Latitude’,’u’:’Lat’,’t’:*reception time,’v’:-296207.45},

 {’n’:’Longitude’,’u’:’Lon’,’t’:60,’v’:-511271.67},

 {’n’:’Velocity’,’u’:’m/s’,’t’:60,’v’:241.92}

 {’n’:’Latitude’,’u’:’Lat’,’t’:60,’v’:-296226.93},

 {’n’:’Longitude’,’u’:’Lon’,’t’:60,’v’:-511312.97},

 {’n’:’Velocity’,’u’:’m/s’,’t’:60,’v’:125.34} ]



### 7.3. Real Time Data

While it is common practice to save IoT data in time series for analysis purposes, there are certain situations where real-time data transmission is necessary. One such scenario is where quick response times are critical. For example, in industrial applications where sensor readings must be acted upon immediately. In these cases, data must be transmitted and analyzed in real-time to ensure the proper functioning of the system.

Then, consider a sensor of temperature sending one measurement using JSON and SenML format.

Listing 49 takes 56 bytes to be transmitted, represented in CBOR only takes 40 bytes, 36 bytes when represented with ASN.1, and 31 bytes when represented with Protocol buffers. Instead, if the VTS representation depicted in Listing 50 is used, only 14 bytes are necessary to represent the same information. Leading to a reduction of the 20% for CBOR and the 75% for VTS, and when compared protocols such as ASN.1 or Protocol buffers with VTS, leads to a reduction of 61% and 54%, respectively.

**Listing 49.** One single measurement of temperature using JSON and SenML format. 

[{’n’:’Temperature’,’u’:’Cel’,’t’:1650830443,’v’:20.45}]



**Listing 50.** Listing 49 in VTS representation. It is important to note, that as the time is not defined the default value in the metadata is assigned (the reception time). 

[{0:1},[{5:’Cel’},2045]]



Additionally, as mentioned in Section 6.2, 18 bytes are necessary to add in the payload which corresponds to the headers from the protocols IP/UDP and CoAP over SCHC, and the headers from LoRaWAN in the MAC and application layer.

**Figure 14:**
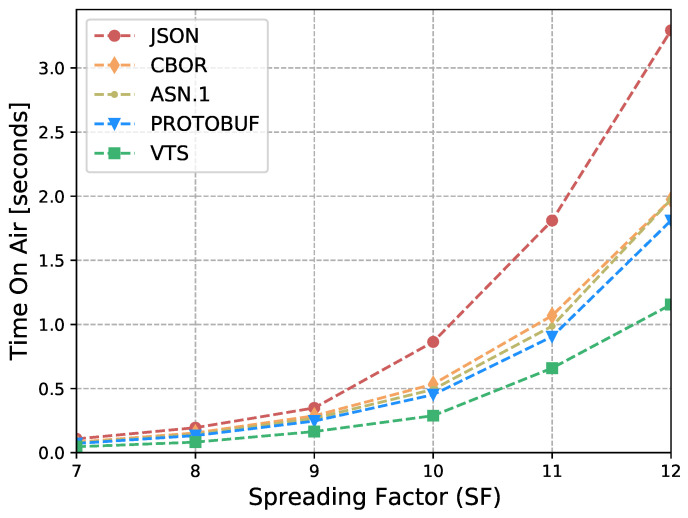
Time-on-Air comparison for real-time data payload per SF.

**Figure 15:**
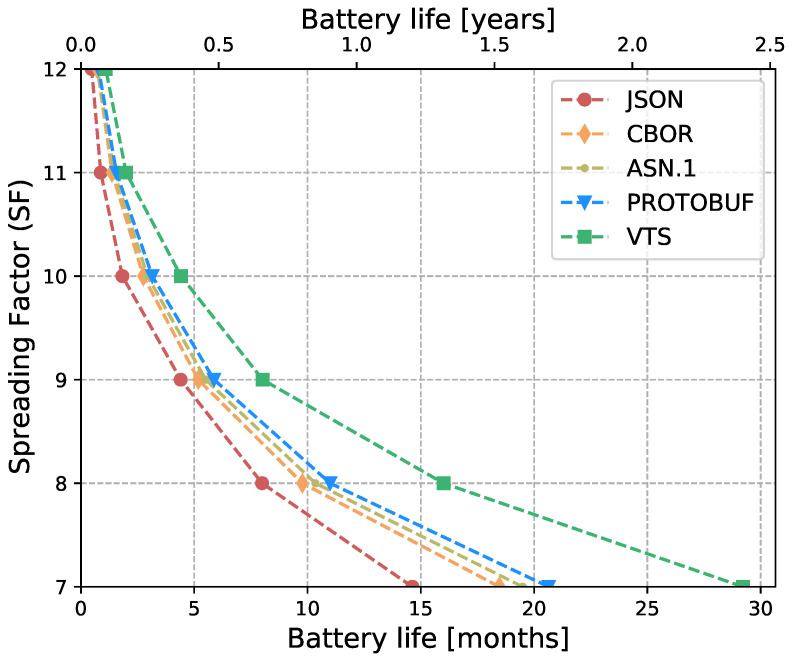
Battery lifetime comparison for real-time data payload per SF.

Consequently, if we consider an application scenario transmitting one package per minute with a battery capacity of ($C_{battery}=2700$ mAh) to calculate the Time-on-Air and Battery Lifetime, we obtain the results depicted in Figure 14 and Figure 15.

When represented in the VTS format, the Time-on-Air is reduced by about 56% (e.g., from 0.107 s to 0.046 s in SF 7) compared to JSON, around 44% (e.g., from 0.082 s to 0.046 s in SF 7) compared to CBOR, around 40% (e.g., from 0.077 s to 0.046 s in SF 7) compared to ASN.1 and around 35% (e.g., from 0.071 s to 0.046 s in SF 7) compared to Protocol buffers. Finally, the Battery Lifetime is increased by around 1.99 times (e.g., from 14.6 months to 29.2 months in SF 7) compared to sending the same information represented in JSON, a 1.7-fold increase (e.g., from 18.4 months to 29.2 months in SF 7) compared to sending the same information represented in CBOR, a 1.5-fold increase (e.g., from 19.5 months to 29.2 months in SF 7) compared to sending the same information represented in ASN.1, and a 1.4-fold increase (e.g., from 20.6 months to 29.2 months in SF 7) compared to sending the same information represented in Protocol buffers.

It is also important to note that while real-time data transmission can be useful in certain scenarios, it is not always necessary. In many cases, time series data storage and analysis are sufficient for identifying trends and making informed decisions. Ultimately, the decision to use real time data transmission versus time series data storage and analysis depends on the specific use case and requirements of the system.

However, if it is necessary to start batching the measurements, the effectiveness of the reduction will gradually increase depending on how many bytes are reduced per measurement in each template, as it varies proportionately to the number of bytes required to represent the difference between measurements.

### 7.4. Transformation into Any Data Format

Our approach aimed to convert the VTS representation, which includes additional data to represent the context of measurements, into any format required by the IoT cloud application. This means that various data formats, such as SenML, JSON, RDF/turtle, RDF/XML, RDF/JSON-LD, or even RDF/EXI can be generated based on the template used and the processing carried out by the proxy in the architecture shown in Section 4.

To achieve the transformation, the template used must be tailored to the desired format. This process enables the data to be transformed into a more suitable format for specific applications. For example, an instance where the data are represented in RDF/XML format is depicted in Listing 7. In order to define the template for this specific format, Listing 51 can be used as a reference.

**Listing 51.** The RDF / XML template representation of the Listing 7. Here the TAGN identify the variables values of the current and voltage.

<?xml version="1.0"?>

<rdf:RDF xmlns:rdf="http://www.w3.org/../../22-rdf-syntax-ns#"

     xmlns:ex="http://...#">


 


  <rdf:Description rdf:about="urn:::0024...804ff1/current">

    <ex:time></ex:time>

    <ex:unit></ex:unit>

    <ex:value>TAGN(0, true)</ex:value>

  </rdf:Description>


 


  <rdf:Description rdf:about="urn:::0024...804ff1/voltage">

    <ex:time></ex:time>

    <ex:unit></ex:unit>

    <ex:value>TAGN(1, true)</ex:value>

  </rdf:Description>


 


</rdf:RDF>



Furthermore, once the template is defined, sending only one CBOR data item in VTS format (as shown in Listing 50) is sufficient to represent the data in Listing 7.

Upon comparing Listing 8 with Listing 52, we can see that the RDF/EXI format repeats the same information in the data as IDs, whereas our proposed approach eliminates the need of sending this information as it is handled in the data transformation process at the proxy side.

**Listing 52.** Listing 49 in VTS representation.

[{0:1,1:1276020074,2:1,3:1},[{5:’Cel’},15,1,1],

[{{5:’V’}}, 1201]]



Finally, it is also important for future research to consider the processing time of data transformation at the proxy side of the architecture.

## 8. Conclusions

As IoT networks expand rapidly, they entail a massive deployment of IoT devices and an incredible amount of data generated by these devices. Interoperability is a major challenge that must be addressed, especially the diversity of devices with distinct data formats. The communication among IoT devices is impeded by the lack of semantic data and common standards for data interpretation, as well as the device incompatibility at the data layer, due to the dynamic nature of the data. Furthermore, the data must be discoverable, which is difficult with enormous volumes of remote networks and cloud-based data. Therefore, this article proposed a novel data format for time series based on CBOR. It enhances the efficiency and interoperability of IoT devices by decoupling the application data format from the sensor data format and reducing the amount of data sent while preserving the same information. We have validated our approach with different data types such as GPS measurements, and have evaluated battery life, Fragmentation, and Time-on-Air to demonstrate its benefits.

The experimental results showed that our approach reduces the actual data sent by IoT devices by between 88% to 94% compared to JSON. It also reduces the Time-on-Air by 84% to 94%, resulting in a 12-fold increase in battery life when compared to the payload in JSON format. Then, when our approach was compared against binary formats such as ASN.1, CBOR, and Protocol buffers, it results in a reduction of the payload of between 54% to 75%, which leads to an increase of battery life of between 1.4 times to 17 times. It should be noted that these results are highly dependent on the implementation of the various binary formats and the nature of the data used in this article. Consequently, other implementations may differ slightly depending on these factors. For example, the results may differ depending on how the schema is selected in ASN.1 or in the type and TAG selection in CBOR.

Moreover, our approach provides a very compact format for time series data that reduces the dependency between cloud applications and IoT devices, since with VTS it is possible to transform to any data format required by the cloud application. Then, our approach can be useful in scenarios where the network bandwidth is limited, allowing for more efficient data transmission and meeting the specific requirements of the target application.

The CDDL code enables the validation of any CBOR structure to check if it follows the rules of the proposed data format for time series. This will facilitate the future transformation of the generated time series into any data format needed by the cloud application by verifying if the structure is well formed or not before proceeding with the transformation process. Furthermore, with the refined metadata, we achieved flexibility in representing different data types and changing the context depending on the application’s needs. However, future work will investigate how to retrieve the context of a packet containing the values when the context is lost.

Finally, we assert that this approach possesses the potential to be presented and deliberated upon within the working groups of the Internet Engineering Task Force (IETF), as a means to initiate a discussion regarding the establishment of a standardized representation for time series utilizing CBOR templates.

## Figures and Tables

**Table 1:** CBOR data types with the corresponding representation and examples.

Data Type	CBOR Representation ^2^	Example ^1^
Unsigned Integer	000 (Major type 0)	345 : 19 0159 hex
Negative Integer	001 (Major type 1)	−345 : 39 0158 hex
Byte String	010 (Major type 2)	“Hello” in ASCII: 4548656C6C6F hex
Text String	011 (Major type 3)	“Hello” as String: 6548656C6C6F hex
Array	100 (Major type 4)	[1,2,3] : 83010203 hex
Map	101 (Major type 5)	{1:5} : A10105 hex
Tag	110 (Major type 6)	Epoch-based date : C11A01020304 hex
Simple/Float Type	111 (Major type 7)	Boolean ’True’ : F5 hex

^1^ All the examples are represented in hexadecimal but the CBOR representation is in binary format. ^2^ The CBOR representation of the data type is identified in the first three bits defined in [16] as a Major type.

**Table 2:** Metadata values.

Name	Meaning	Value Defined	Unit	Default Value	Type ^1^	Example
**Context**	Specific number which indicates the other values of the metadata.	0	Integer	0	G	{0:23}
**Time**	Time when the first measurement in the CBOR data item was taken.	1	EPOCH time	Reception time	G & S	{1:16,540,740}
**Time difference**	Difference in time with the subsequent values.	2	Seconds	60	G & S	{2:30}
**Precision**	Decimal component of a floating-point number to convert into an integer.	3	Integer	2	G & S	{3:1}
**Delta**	Difference between the subsequent values.	4	1, 2 or 10	1	G & S	{4:23}
**Unit**	Metric unit used for each variable taken.	5	String, all listed in [42].	–	S	{5:‘Cel’}

^1^ The type corresponds to where it is possible to find the metadata value, in the general metadata (G), specific metadata (S) or both (G & S).

**Table 3:** Maximum payload size per SF in LoRaWAN for the EU863-870 MHz Band [48].

Spreading Factor (SF)	Payload Size (Bytes)
12	59
11	59
10	59
9	123
8	230
7	230

**Table 4:** Current per state for transceiver Semtech SX1272.

State	Current ($I_{i}$) mA
Rx	11.2
Tx	125
Sleep	0.001
Standby	1.4

**Table 5:** Metadata comparison between actual proposal and proposed in [17].

	Metadata Defined in [17]			New Definition of Metadata		
	**LoRaWAN**	**Wi-Fi**	**LTE/5G**	**LoRaWAN**	**Wi-Fi**	**LTE/5G**
**Packets number**	10	2	2	10	2	2
**Size in Bytes**	170	34	34	40	16	16
**Percentage (%)**	1.9	0.4	0.4	0.5	0.2	0.2

## Data Availability

Publicly available datasets were analyzed in this study. These data can be found here: [https://github.com/sebasmol96/Time-Series-data-set-for-CBOR-templates]. (accessed on 1 March 2023).
